# Unique amino acid substitution within the spike glycoprotein S1 subunit promotes infectious bronchitis virus infection via enhancing viral attachment

**DOI:** 10.1128/mbio.00884-26

**Published:** 2026-09-03

**Authors:** Tianyi Liu, Yujuan Zhang, Zongxi Han, Huixin Li, Yuhao Shao, Junfeng Sun, Shengwang Liu

**Affiliations:** 1 Division of Avian Infectious Diseases, State Key Laboratory of Animal Disease Control and Prevention, Harbin Veterinary Research Institute, the Chinese Academy of Agricultural Sciences 111613, Harbin, People's Republic of China; Tsinghua University, Beijing, China

**Keywords:** infectious bronchitis virus, circular polymerase extension reaction, S1 subunit, attachment, cell tropism

## Abstract

Due to the scarcity of cell-adapted strains and limitations of current reverse genetics techniques, the factors determining cell tropism of infectious bronchitis virus (IBV), a prototype *gammacoronavirus*, remain unclear. Here, we demonstrated that the expanded cell tropism of CEA, a chicken embryo fibroblast-adapted strain derived from tl/CH/LDT3/03, was related to its enhanced attachment capacity. Then, a reverse genetics platform for IBV based on circular polymerase extension reaction (CPER) was established, enabling the successful recovery of rCEA and rLDT, which retained the biological properties of their respective parental viruses. Using this platform, we constructed a series of chimeric viruses based on the CEA strain by incorporating the entire S gene, the S1 and S2 subunits, and substitutions at differential sites derived from the tl/CH/LDT3/03 strain. Our findings indicated that subunit S1, rather than S2, was associated with viral adaptation in DF-1 cells, with the amino acid residue at position 413 in the C-terminal domain of the S1 protein as a key determinant. Evaluation with recombinant S1 proteins and chimeric viruses revealed that the histidine residue at position 413 (His413) promoted enhanced attachment of both S1 proteins and viruses to cells, while exerting no direct effect on viral entry. Mechanistically, His413 facilitated efficient viral binding to α-2,3-linked sialic acids presented on gangliosides rather than glycoproteins. This interaction activated Src kinase and triggered caveolae-mediated endocytosis, initiating viral entry and subsequent replication. Collectively, the CPER-based reverse genetics platform established in this study represents a significant technical advancement for IBV, and our findings provide novel insights into the cell adaptation and entry mechanisms of IBV.

**IMPORTANCE:**

Although infectious bronchitis virus (IBV) was the first discovered coronavirus, the cellular receptors and cofactors that mediate successful infection, as well as the specific pathways and mechanisms of viral entry into host cells, remain to be elucidated. In this study, we established a reverse genetics platform for IBV using circular polymerase extension reaction for the first time. A series of chimeric viruses were subsequently recovered, and a specific amino acid substitution at position 413 in the C-terminal domain of the S1 subunit was identified as a key determinant for the expanded DF-1 cell tropism of IBV. The histidine residue at this site facilitated viral binding to α-2,3-linked sialic acids on gangliosides, thereby activating the caveola-mediated endocytosis and enabling viral entry into DF-1 cells. This study provides novel insights into the receptor-binding function of the IBV S protein and the strategies employed by the virus for cell adaptation. Additionally, our findings offer new perspectives for developing cell-culture-based vaccines.

## INTRODUCTION

Infectious bronchitis virus (IBV), a prototype *gammacoronavirus* within the family *Coronaviridae*, order *Nidovirales*, infects the respiratory tract, urinary system, and reproductive tract of chickens, causing substantial economic losses to the poultry industry worldwide (1, 2). Generally, IBV isolates exhibit restricted cell and tissue tropism. Though some IBV strains, following adaptation, can replicate in primary chicken kidney (CK) cells and chicken embryo kidney (CEK) cells, the vast majority of IBV isolates are capable of replicating only in embryonated chicken eggs (3–6). To date, only a few strains, including Beaudette, HV80, VEA P25, and CEA P20, have demonstrated the ability to replicate efficiently in certain cell lines cultured *in vitro* following serial passages in embryonated eggs or cells (7–10).

Entry into host cells is an essential step for IBV to establish infection and is mediated by the spike (S) glycoprotein on the viral envelope surface (5). The S glycoprotein is synthesized as a precursor and cleaved in host cells to form the non-covalently associated S1 and S2 subunits. The S1 subunit, responsible for receptor binding, and the S2 subunit, which mediates membrane fusion, are both indispensable for viral infection (11, 12). Several specific amino acid residues within the S2 subunit, primarily located in the S2′ motif, have been demonstrated to be associated with the extended cellular tropism of IBV strains Beaudette and HV80 for Vero cells, SczyC30 for CEK cells, and H120 for CK cells (13–16). It was speculated that these residues may facilitate viral infection by promoting the occurrence of virus–cell membrane fusion. Additionally, the unique motif (RRKR/S) present in the S2′ site of the Beaudette strain enables it to utilize heparan sulfate (HS) as an attachment receptor to facilitate viral infection and may also promote viral entry and syncytium formation in a furin-dependent manner (17, 18). It should be noted that among the numerous IBV isolates, the S2′ site with this unique motif is presented only in two IBV strains, Beaudette and HV80. In contrast, it is absent in two other reported cell-adapted strains, VEA P25 and CEA P20, as well as in strain 250607GXCX, which is capable of infecting Vero and DF-1 cells without adaptation in eggs or cells (19). This suggests that, in addition to S2-mediated virus–cell membrane fusion, other viral factors are also involved in the cell tropism of IBV. Recently, our team obtained the CEA P20 strain through adaptation of the tl/CH/LDT3/03 strain (LDT3/03) in chicken embryo fibroblast (CEFs) (9). Although both strains share an identical S2′ motif and neither possesses the RRKR/S sequence, only CEA P20 replicates efficiently in CEFs, making it an ideal viral model for in-depth investigation into the determinants of IBV cell tropism.

The receptor-binding domain (RBD) of coronaviruses (CoVs) is located in the S1 subunit. Based on the function and structure annotations, the S1 subunit of CoVs can be further divided into two domains: the N-terminal domain (NTD) and the C-terminal domain (CTD), which are considered to be involved in the recognition of carbohydrates and transmembrane protein receptors, respectively (12, 20–22). Unlike *alphacoronaviruses* and *betacoronaviruses*, for which specific protein receptors have been identified, the authentic protein receptor for *gammacoronaviruses,* including IBV, has yet to be discovered (21, 23). It is currently known that only HS and sialic acids can serve as attachment factors to facilitate IBV infection. Specifically, HS is solely utilized by the Beaudette strain that contains the unique S2′ motif (17), whereas sialic acids are generally involved in IBV infection both *in vitro* and *in vivo* (24–29). Structural resolution of the IBV S protein reveals that the NTD and CTD of the S1 subunit each contain a putative RBD (30). The sialic acid-binding domain of certain IBV strains has been mapped within a region of approximately 200 amino acids starting from the 19th amino acid at the N-terminus within S1-NTD. It has been demonstrated that specific amino acid substitutions in this region can alter the binding capacity of IBV for sialic acids, thereby leading to changes in viral tissue tropism *in vivo* (25, 26, 29). In contrast, although the CTD of S1 is predicted to contain an RBD and is implicated in IBV entry into chicken fibroblasts (30), the molecular mechanism underlying its receptor-binding properties and its contribution to the cell tropism of IBV has yet to be elucidated. Whether amino acid substitutions in the S1-CTD, similar to those in the NTD, can modulate receptor-binding capacity, thereby altering viral cell tropism independently of S2-mediated membrane fusion remains to be further elucidated. Upon binding to receptors or attachment factors, CoVs can either fuse directly at the cell surface or enter the cell via the endocytic pathway, where membrane fusion is initiated by the acidic pH conditions and certain proteases within the endosomal compartments (31). Current understanding of the entry pathways utilized by IBV is primarily derived from studies on the Beaudette strain, which can enter various cell types, including Vero, Huh7, DF-1, and H1299 cells, via clathrin-mediated endocytosis (CME). Additionally, this strain can also utilize caveola-mediated endocytosis (CavME) to enter H1299 cells (32). These findings suggest that IBV may exploit alternative pathways to enter into different cells. However, little information is available regarding the detailed entry mechanism of IBV.

Another limiting factor hindering the elucidation of IBV gene function and pathogenicity is the lack of reliable reverse genetics systems. Due to its large genome size of approximately 27 kb, the construction of full-length cDNA poses the greatest challenge in the development of reverse genetics platforms for IBV. Currently, some strategies, such as the vaccinia virus-based system (33), *in vitro* ligation (34), the targeted RNA recombination technique (35), the bacterial artificial chromosome system (36), and the transformation-associated recombination technology (37), have been employed for the development of the IBV reverse genetics platform. While these strategies have been successfully applied to the rescue of several IBV strains, the process remains limited by reliance on specific restriction sites, the instability of gene fragments in bacterial systems, and the technical complexity and time-consuming nature of the procedures. Recently, a bacterium-free circular polymerase extension reaction (CPER) system, originally designed to construct infectious cDNA of flaviviruses (38), has been successfully applied to the rescue of SARS-CoV-2 (39, 40). This system enables rapid and efficient assembly of the large genome of CoVs and facilitates convenient genetic manipulation, thereby providing an attractive method for the rescue of IBV.

In this study, we demonstrated that the enhanced attachment capacity of the IBV CEF-adapted strain CEA played a key role in enabling its expanded cellular tropism and efficient propagation in multiple chicken-origin cells. To elucidate the molecular basis, we established, for the first time, a CPER-based reverse genetics system for IBV. The recovered viruses, rLDT and rCEA, retained the biological properties of their respective parental viruses. Using the established system, we successfully rescued a series of viruses carrying chimeric or single-site-substituted S proteins and further demonstrated that the amino acid residue at position 413 in the CTD of the S1 subunit was a critical determinant of viral cell tropism and replication. The histidine residue at this site mediated efficient binding of virus to α-2,3-linked sialic acids located at the termini of gangliosides, thereby triggering Src activation and facilitating viral entry into cells via CavME.

## RESULTS

### Cell infection characteristics of LDT3/03 P3 and CEA P23

**Fig 1:**
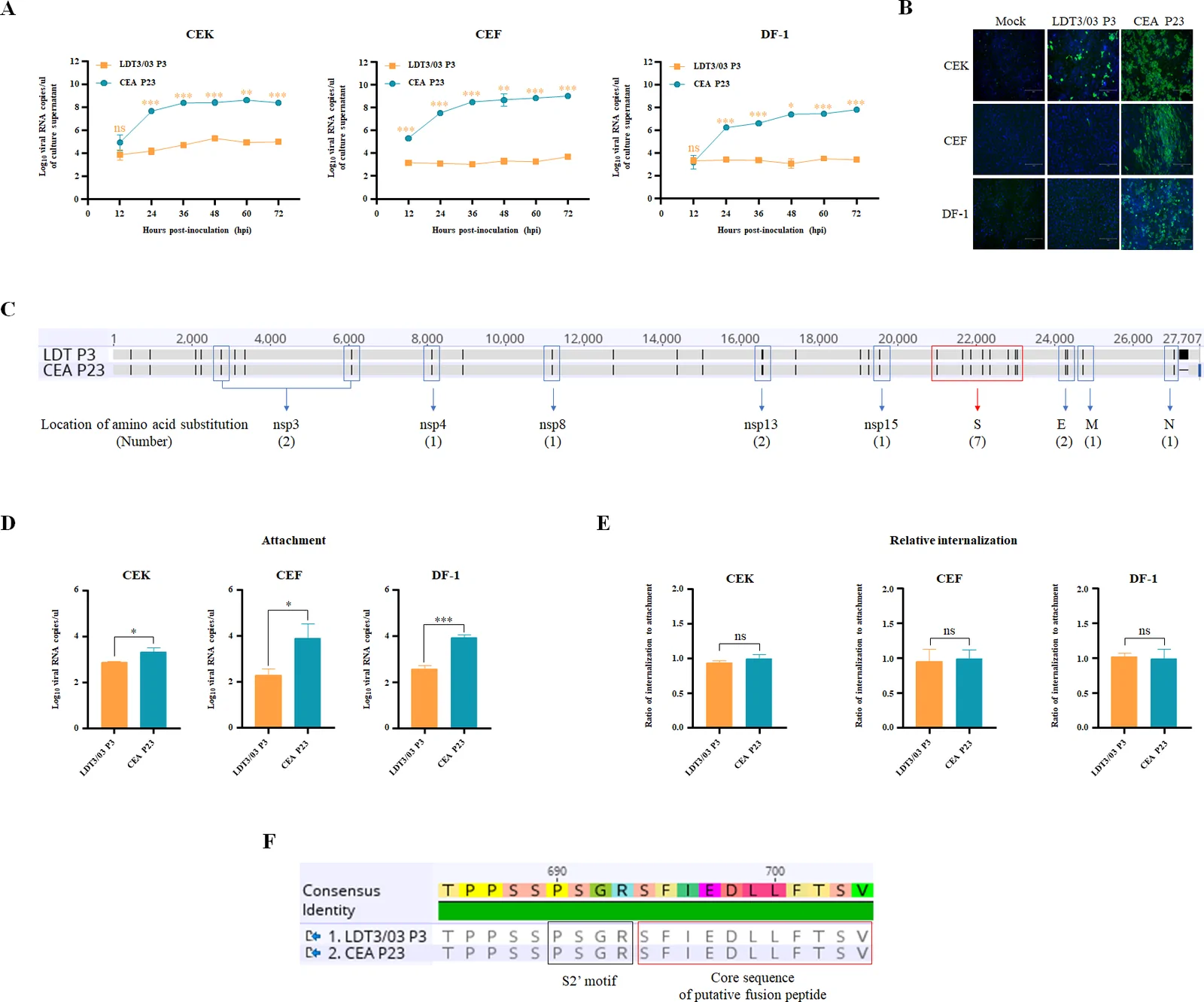
Cell infection characteristics of LDT3/03 P3 and CEA P23 in multiple chicken-origin cells. (**A and B**) Growth features of LDT3/03 P3 and CEA P23. CEK, CEF, and DF-1 cells were inoculated with each virus at a dose of 5 × 10^7^ viral RNA copies. Viral RNA in supernatants was extracted and quantified by real-time RT-PCR at the indicated time points (**A**). At 36 hpi, cells were fixed and immunostained for the IBV N protein (green), and nuclei were counterstained with DAPI (blue). Scale bars, 150 µm (**B**). (**C**) The sequence differences between LDT3/03 P3 and CEA P23. Whole-genome sequences were aligned using Geneious Prime software, and the differential nucleotides were presented as black lines. Amino acid substitutions in specific viral proteins in the CEA strain were shown below. (**D and E**) The attachment and internalization capacities of LDT3/03 P3 and CEA P23. CEK, CEF, and DF-1 cells were inoculated with each virus at a dose of 5 × 10^9^ viral RNA copies and then subjected to attachment (**D**) and internalization (**E**) assays. Attached and internalized viral RNAs were extracted and quantified by real-time RT-PCR. Internalization efficiency was calculated as the ratio of internalized to attached viral RNA. (**F**) Alignment of the S2′ motif and core sequences of the putative fusion peptide between LDT3/03 P3 and CEA P23. Data were presented as means ± SD, and statistical significance was determined using two-way ANOVA with Sidak’s multiple comparisons test (**A**) or unpaired two-tailed Student’s *t*-test (**D and E**) (*n* = 3 biologically independent samples). *P < 0.05; **P < 0.01; ***P < 0.001; ns, no significant difference.

To investigate the replication features of LDT3/03 P3 and CEA P23, the growth kinetics of both viruses in CEK, CEF, and DF-1 cells were first determined. As shown in Fig. 1A, a limited increase in RNA copies of LDT3/03 P3 was observed only in the supernatant of CEK cells from 12 to 72 h post-inoculation (hpi), while viral RNA copies of this virus in the supernatant of both CEF and DF-1 cells remained at a relatively constant level with time. Comparatively, the significantly increased viral RNA copies within 72 hpi indicated that CEA P23 could effectively propagate in all three types of cells. The propagation features of LDT3/03 P3 and CEA P23 were further examined via indirect immunofluorescence assay (IFA) using an antibody against the viral N protein. Consistent with the viral RNA detection results, sporadic fluorescence was observed only in LDT3/03 P3-inoculated CEK cells at 36 hpi, while fluorescence was negligible in both LDT3/03 P3-inoculated CEF and DF-1 cells. In contrast, obvious fluorescence was observed in all three types of cells inoculated with CEA P23 (Fig. 1B). Moreover, the IFA results indicated that CEA P23 was capable of replicating in Vero cells, whereas LDT3/03 P3 was not (Fig. S1). These data indicated that compared with LDT3/03 P3, CEA P23 exhibited expanded cell tropism and could efficiently replicate in multiple chicken-origin cells as well as Vero cells.

Our recent findings revealed that most of the amino acid differences between LDT3/03 and CEA strains were located within the S protein (9), with a small number of additional amino acid substitutions presented in several other structural and non-structural proteins (Fig. 1C). Since S protein is recognized as a key determinant of CoVs cell tropism and is directly responsible for the cellular entry process of CoVs (41, 42), the attachment and internalization capacities of LDT3/03 P3 and CEA P23, which are closely related to the function of the S protein, were further evaluated. For viral attachment, CEK, CEF, and DF-1 cells were incubated with both viruses, LDT3/03 P3 and CEA P23, for 1 h at 4°C. RNA from attached virus was extracted and quantified by real-time RT-PCR. As shown in Fig. 1D, CEA P23 demonstrated significantly higher viral RNA copies than LDT3/03 P3, indicating the superior attachment capacity of CEA P23 across three chicken-origin cells. Furthermore, CEK, CEF, and DF-1 cells attached to LDT3/03 P3 and CEA P23 were further incubated at 37°C for 1 h to permit internalization, and then the internalization efficiency was calculated by normalizing the RNA from internalized virus to that from attached virus. The results demonstrated that there was no significant difference between LDT3/03 P3 and CEA P23 in terms of internalization capacity among three types of cells (Fig. 1E). Of note, the S2′ motif and core sequences of putative fusion peptide in S proteins of LDT3/03 P3 and CEA P23, which are responsible for the membrane fusion between IBV and host cell, were identical (Fig. 1F). Collectively, these findings suggest that the enhanced attachment capacity of CEA P23, mediated by the S protein, may contribute to its expanded cell tropism.

### Establishment of a reverse genetics system for IBV using CPER

**Fig 2:**
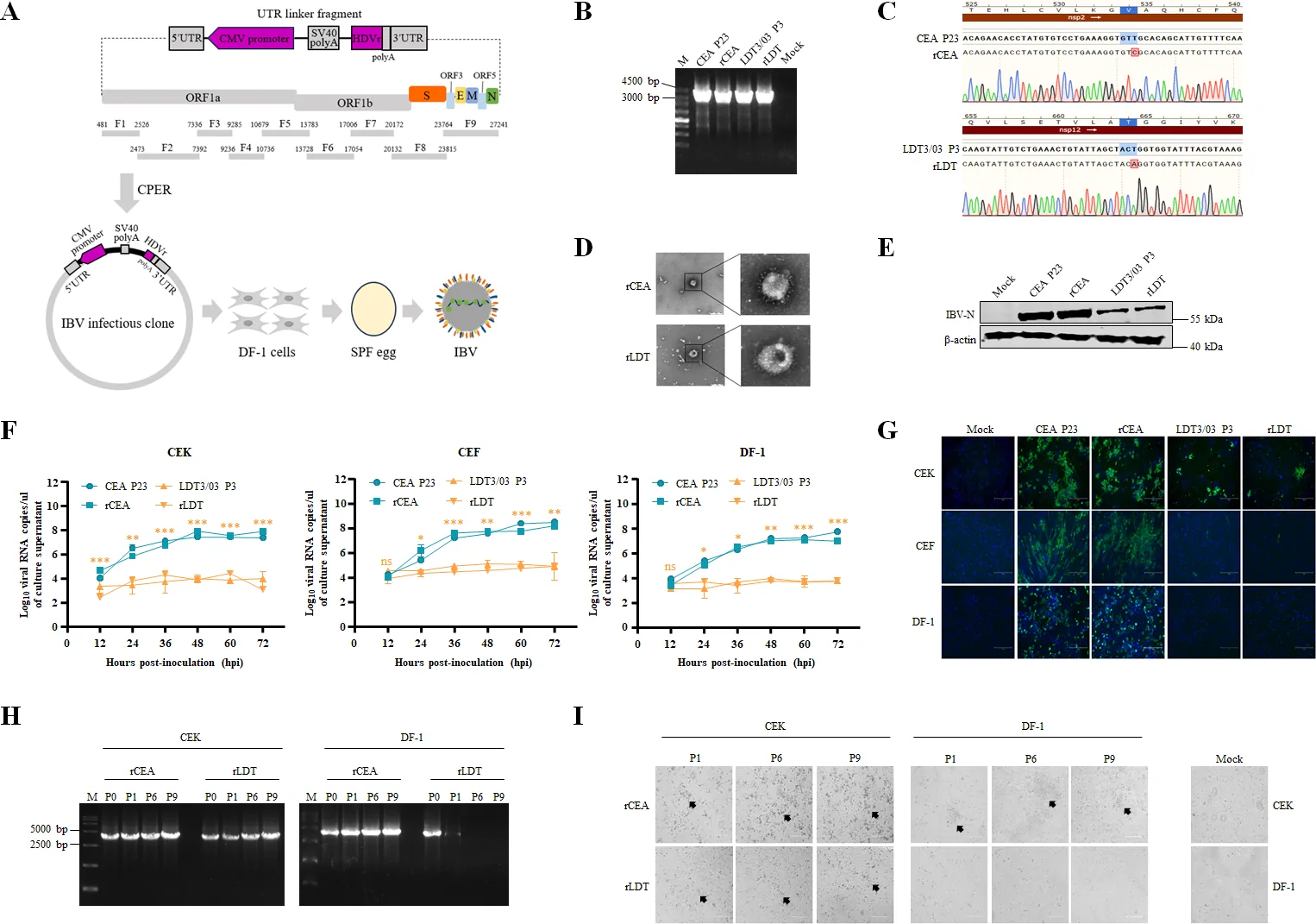
Establishment of a CPER-based reverse genetics system for IBV. (**A**) Schematic diagram of the CPER strategy for generating infective IBV. Ten overlapping fragments covering the entire genome of the virus, along with UTR linkers, were amplified and assembled via CPER. The resulting circular products were transfected into DF-1 cells, and culture supernatants as well as transfected cells were subsequently inoculated into 9-day-old SPF embryonated chicken eggs for virus propagation. (**B–E**) Identification of recovered viruses, rCEA and rLDT. The presence of the G8 fragment (3,684 nt) (**B**) and the signature genetic markers T2173C (for rCEA) and T14382A (for rLDT) (**C**) were confirmed by RT-PCR and sequencing. Viral particle morphology was visualized by transmission electron microscopy (**D**). Expression of the viral N protein in virus-infected CEK cells at 36 hpi was detected by Western blotting (**E**). (**F and G**) Comparison of growth features between parental and recovered viruses. CEK, CEF, and DF-1 cells were inoculated with the parental strains CEA P23 and LDT3/03 P3, as well as recovered viruses rCEA and rLDT at a dose of 5 × 10^7^ viral RNA copies. Viral RNA in supernatants was extracted and quantified by real-time RT-PCR at the indicated time points (**F**). At 36 hpi, cells were fixed and immunostained for N protein (green), and nuclei were counterstained with DAPI (blue). Scale bars, 150 µm (**G**). (**H and I**) Genetic stability of recovered viruses upon serial passage in CEK and DF-1 cells. rCEA and rLDT harvested from allantoic fluid (designated P0) were serially passaged nine times in both CEK and DF-1 cells, respectively. Viral RNA was extracted from cell culture supernatants collected at passages 1 (P1), 6 (P6), and 9 (P9), and the presence of the G8 fragment (3,684 nt) was assessed by RT-PCR (**H**). Representative images of virus-infected cells were shown; black arrows indicated cytopathic effects (CPEs) induced by rCEA in both CEK and DF-1 cells, and by rLDT only in CEK cells, at the indicated passages (**I**). Data were presented as means ± SD, and statistical significance was determined using two-way ANOVA with Sidak’s multiple comparisons test (**F**) (*n* = 3 biologically independent samples). *P < 0.05; **P < 0.01; ***P < 0.001; ns, no significant difference.

To elucidate the molecular basis underlying the expanded cellular tropism of IBV, a reverse genetics system for virus rescue was established using CPER as described previously (40). As illustrated in Fig. 2A, nine overlapping viral gene fragments covering the entire genome of CEA P23 or LDT3/03 P3 and a UTR linker fragment containing genomic 3′ terminal sequence, hepatitis delta virus ribozyme (HDVr) sequence, simian vacuolating virus 40 (SV40) poly A signal, cytomegalovirus (CMV) promoter sequence, and genomic 5′ terminal sequence were amplified and subsequently assembled into full-length cDNA clones via CPER. The unpurified CPER products were directly co-transfected into DF-1 cells with helper plasmid pCAG-N, following inoculation into 9-day-old specific pathogen-free (SPF) embryonated chicken eggs for propagation. Stock of each recovered virus was prepared by inoculating SPF eggs once again. The obtained viruses were named rCEA and rLDT, respectively.

Stocks of rCEA and rLDT were first examined via RT-PCR with the primer pair G8, with parental strains CEA P23 and LDT3/03 P3 included as controls. As expected, genomic fragment G8 was successfully amplified from all four viruses (Fig. 2B). Besides, the genomic regions of rCEA and rLDT containing the introduced genetic markers were amplified, and the presence of introduced synonymous mutations, T2173C in rCEA and T14382A in rLDT, was confirmed by sequencing (Fig. 2C). Subsequently, virus particles with typical coronavirus morphology were observed in stocks of rCEA and rLDT by electron microscopy (Fig. 2D). Moreover, CEK cells were inoculated with rCEA, rLDT, and their respective parent viruses. The expression of the N protein in each virus-infected CEK cells was confirmed by Western blotting (Fig. 2E).

Next, the replication features of recovered rCEA and rLDT were evaluated. The growth kinetics of the two recovered viruses and their biological parents in CEK, CEF, and DF-1 cells were first determined. As shown in Fig. 2F, similar to parental CEA P23 and LDT3/03 P3, rescued rCEA could replicate efficiently in these three types of cells, whereas rLDT replicated only in CEK cells. As expected, growth kinetics of rCEA and rLDT in each type of cell were comparable with those of CEA P23 and LDT3/03 P3, respectively (Fig. 2F). Replication phenotypes of these four viruses in CEK, CEF, and DF-1 cells were further verified via IFA targeting viral N protein (Fig. 2G). To assess the genetic stability of recovered viruses, rCEA and rLDT were serially passaged nine times in both CEK and DF-1 cells. The presence of viruses in the supernatant of passages 1, 6, and 9 in each cell type was examined by RT-PCR with the primer pair of G8. As shown in Fig. 2H and I, after nine consecutive passages in CEK cells, genomic RNA from both rCEA and rLDT was detectable, and CPEs induced by viral infection were consistently observed. However, after serial passages in DF-1 cells, only rCEA exhibited detectable genomic RNA and was able to induce CPEs. These results demonstrate that the CPER-based reverse genetics system for IBV is a reliable platform for recovering viruses that retain the biological properties of their parental viruses.

### Residue 413 in the S1 subunit contributes substantially to IBV replication in cells

**Fig 3:**
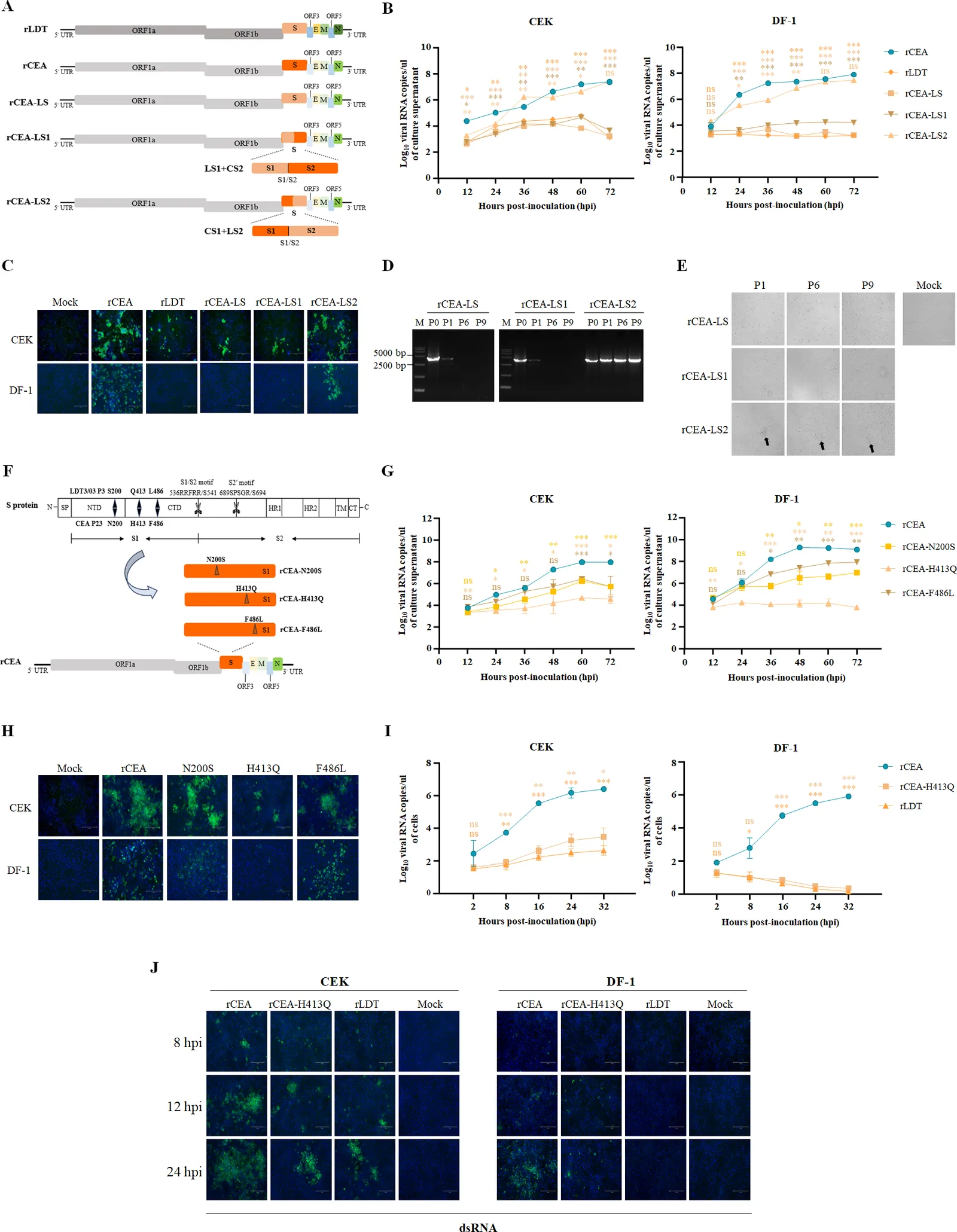
Residue 413 in the S1 subunit is a key determinant of IBV replication capacity in cells. (**A**) Schematic diagram of chimeric viruses rCEA-LS, -LS1, and -LS2, in which the S, S1, and S2 subunits of rCEA were replaced with the corresponding region from LDT3/03 P3, respectively. (**B and C**) Comparison of growth features of these chimeric viruses. Both CEK and DF-1 cells were inoculated with rCEA, rLDT, rCEA-LS, -LS1, and -LS2, and viral RNA in supernatants collected at the indicated time points was quantified as described in Fig. 2F (**B**). At 36 hpi, cells were immunostained for N protein as described in Fig. 2G. Scale bars, 150 µm (**C**). (**D and E**) Genetic stability of rCEA-LS, -LS1, and -LS2 upon serial passage in DF-1 cells. These three chimeric viruses were serially passaged in DF-1 cells, and the presence of the G8 fragment was assessed by RT-PCR as described in Fig. 2H (**D**). Representative images of CPEs (indicated with black arrows) induced by rCEA-LS2 at the indicated passages were shown (**E**). (**F**) Schematic representation of mutants rCEA-N200S, -H413Q, and -F486L, in which the residues at positions 200, 413, and 486 were replaced with the corresponding residues from LDT3/03 P3, respectively. S glycoprotein domains and differential amino acid sites were indicated. SP, signal peptide; NTD, N-terminal domain; CTD, C-terminal domain; HR1, heptad repeat 1; HR2, heptad repeat 2; TM, transmembrane domain; CT, cytoplasmic tail. (**G and H**) Evaluation of growth features of these mutant viruses. Both CEK and DF-1 cells were inoculated with rCEA, rCEA-N200S, -H413Q, and -F486L, and viral RNA in supernatants collected at the indicated time points was quantified as described in Fig. 2F (**G**). At 36 hpi, cells were immunostained for N protein. Scale bars, 150 µm (**H**). (**I and J**) Comparison of the levels of total viral RNA and dsRNA produced during the replication process of rCEA, rCEA-H413Q, and rLDT. Both CEK and DF-1 cells were inoculated with each virus, collected at the indicated time points, and subjected to viral RNA extraction and quantification by real-time RT-PCR (**I**) and detection of dsRNA (green) via the immunofluorescence assay (**J**). Scale bars, 150 µm. Data were presented as means ± SD, and statistical significance was determined using two-way ANOVA with Sidak’s multiple comparisons test (B, G, and I) (*n* = 3 biologically independent samples). *P < 0.05; **P < 0.01; ***P < 0.001; ns, no significant difference.

Given that the cell tropism of IBV is determined by the S protein and that the amino acid differences between CEA and LDT3/03 strains are primarily located within this viral protein (9, 41), the coding sequences (CDSs) of the whole S gene, S1, and S2 subunits of CEA P23 were replaced with those of LDT3/03 P3, respectively. Three corresponding chimeric viruses, rCEA-LS, -LS1, and -LS2, were recovered using the reverse genetic system described above (Fig. 3A), and successful rescue was confirmed via RT-PCR and Western blotting as previously described (Fig. S2A and B). Besides, the S gene sequences of rCEA-LS, -LS1, and -LS2 were confirmed by sequencing (Fig. S2C). Subsequently, growth kinetics of recovered viruses were determined in CEK and DF-1 cells, respectively. As shown in Fig. 3B, apart from rCEA, only the rCEA-LS2, which retained the rCEA S1 subunit, demonstrated a significant increase in viral RNA copies in the supernatant of CEK cells over time and reached viral RNA copies comparable to those of rCEA after 48 hpi. In contrast, only a low-level increase in viral RNA copies was detected in the supernatant of CEK cells inoculated with rCEA-LS and -LS1, similar to rLDT. Besides, similar replication kinetics of rCEA and rCEA-LS2 were observed in DF-1 cells, while no obvious increase in viral RNA copies was detected in the supernatant of DF-1 cells inoculated with rCEA-LS or rCEA-LS1, indicating that these two viruses failed to replicate in DF-1 cells. The differences in replication capacity of these chimeric viruses in both CEK and DF-1 cells were further verified by IFA. At 36 hpi, robust fluorescence of the N protein was visualized in both CEK and DF-1 cells inoculated with either rCEA or rCEA-LS2. In contrast, only sporadic fluorescence was observed in CEK cells inoculated with rLDT, rCEA-LS, or rCEA-LS1. The fluorescence was not visible in these three virus-inoculated DF-1 cells (Fig. 3C). Furthermore, after nine serial passages in DF-1 cells, only rCEA-LS2 was found to retain detectable genomic segments in the supernatants and could induce CPEs upon inoculation into DF-1 cells (Fig. 3D and E). Taken together, these results mapped a determinant of cell tropism of CEA P23 to the viral S1 subunit.

Comparative analysis of the S1 subunit sequences between CEA P23 and LDT3/03 P3 identified three amino acid differences at positions 200, 413, and 486. In the S1 subunit of CEA P23, the amino acid residues at positions 200, 413, and 486 were asparagine (Asn), histidine (His), and phenylalanine (Phe), whereas those in the LDT3/03 P3 were serine (Ser), glutamine (Gln), and leucine (Leu), respectively. Thus, Asn200Ser, His413Gln, and Phe486Leu substitutions were introduced into the S1 of CEA P23, and three corresponding viruses, rCEA-N200S, -H413Q, and -F486L, were recovered (Fig. 3F). Successful rescue of these viruses was verified via RT-PCR and Western blotting as previously described (Fig. S2D and E). The S gene sequences of rCEA-N200S, -H413Q, and -F486L were confirmed by sequencing (Fig. S2F). The growth kinetics of rCEA, rCEA-N200S, -H413Q, and -F486L in both CEK and DF-1 cells were determined to compare the replication capacities of these viruses. Compared with rCEA, all three mutants exhibited significantly reduced replication in both cell types, with the most pronounced decline observed for rCEA-H413Q (Fig. 3G). The N protein expression detected by IFA at 36 hpi in both CEK and DF-1 cells inoculated with rCEA, rCEA-N200S, -H413Q, and -F486L further confirmed this result (Fig. 3H).

To further evaluate the contribution of residue 413 in the S1 subunit to the differential replication phenotypes, total viral RNA and dsRNA levels were assessed in both CEK and DF-1 cells inoculated with rCEA, rCEA-H413Q, or rLDT. Comparison of the growth kinetics of rCEA, rCEA-H413Q, and rLDT in both CEK and DF-1 cells revealed that the sole substitution at position 413 in the S1 subunit was sufficient to reduce the total viral RNA level of rCEA within cells to a level comparable to that of rLDT (Fig. 3I). Furthermore, the detection of dsRNA generated during the replication of the viral genomic RNA showed that progressively more prominent dsRNA signals were observed in rCEA-inoculated CEK cells over time. In contrast, only low levels of dsRNA were detected in cells inoculated with rLDT. Notably, the dsRNA levels in rCEA-H413Q-inoculated cells were comparable to those observed in the rLDT group. This phenomenon was even more pronounced in DF-1 cells (Fig. 3J). Collectively, these results suggest that the amino acid residue at position 413 in the S1 subunit contributes substantially to the observed differences in cell tropism and replication capacity between CEA P23 and LDT3/03 P3.

### His413 in the S1 subunit promotes enhanced attachment of IBV

**Fig 4:**
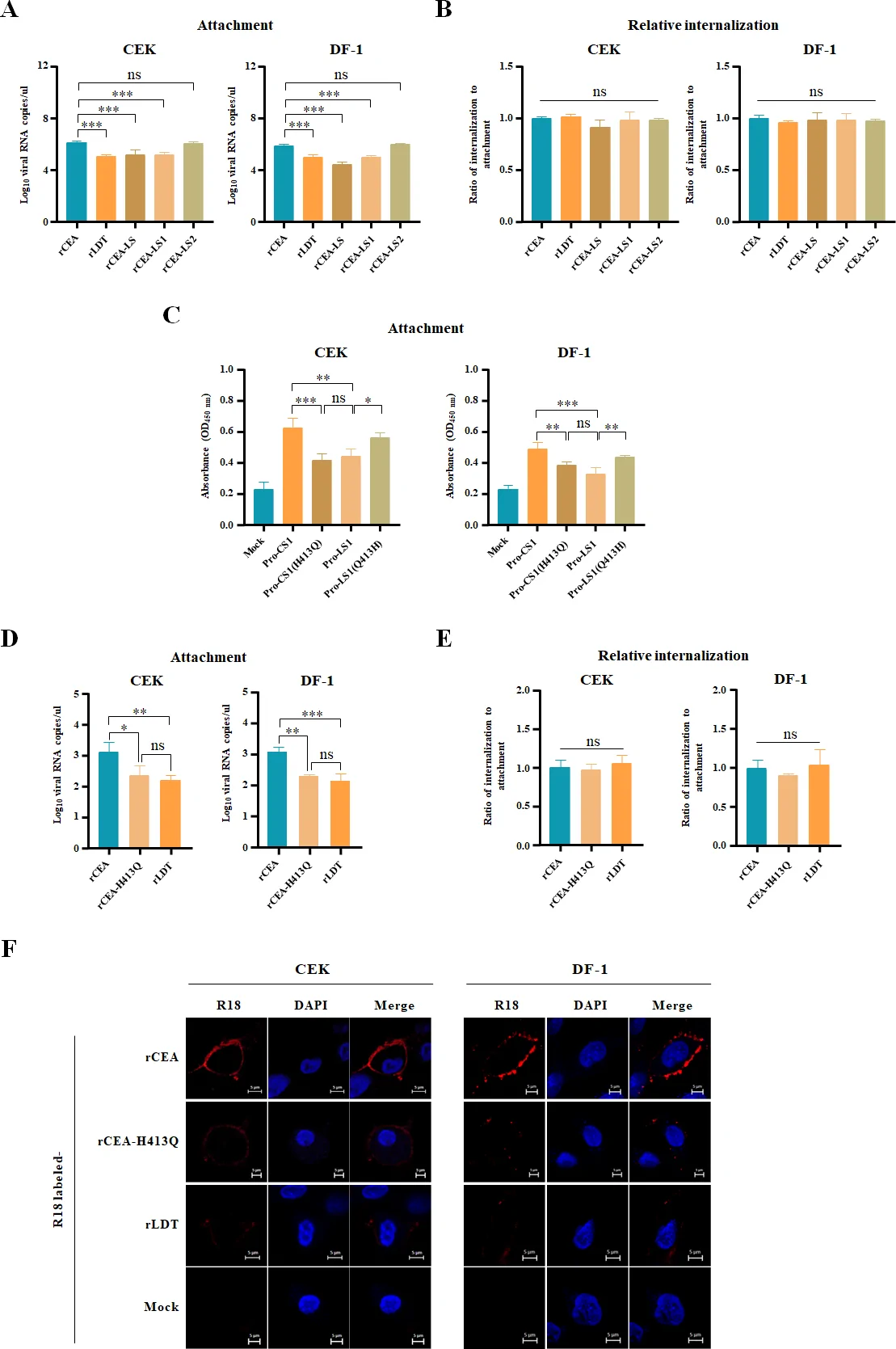
Residue His413 within the S1 subunit mediates the efficient attachment of IBV. (**A and B**) Comparison of the attachment and internalization capacities of rCEA, rLDT, rCEA-LS, -LS1, and -LS2. Both CEK and DF-1 cells were inoculated with the indicated viruses at a dose of 5 × 10^9^ viral RNA copies and subjected to attachment (**A**) and internalization (**B**) assays. Attached and internalized viral RNAs were extracted and quantified by real-time RT-PCR. Internalization efficiency was calculated as the ratio of internalized to attached RNA. (**C**) Evaluation of the binding ability of wild-type S1 proteins and mutants carrying amino acid substitutions at position 413 to cells. Paraformaldehyde-fixed CEK and DF-1 cells were incubated with the indicated C-terminally Strep-tagged S1 proteins for 2 h at 4°C. Cell surface-bound proteins were detected using an HRP-conjugated anti-Strep antibody, followed by tetramethylbenzidine (TMB) substrate development. Absorbance was measured at 450 nm after stopping the reaction with 0.5 M H₂SO₄. (**D and E**) Comparison of the attachment and internalization capacities of rCEA, rCEA-H413Q, and rLDT. Attachment (**D**) and internalization (**E**) assays were performed with the indicated viruses as described in panels **A** and **B**, respectively. (**F**) Examination of viral attachment via confocal microscopy. Both CEK and DF-1 cells were inoculated with R18-labeled rCEA, rCEA-H413Q, and rLDT at 4°C for 1 h. Nuclei were stained with DAPI (blue). The attached virions (red) on the cell surface were visualized by confocal microscopy. Scale bars, 5 μm. Data were presented as means ± SD, and statistical significance was determined using one-way ANOVA with Bonferroni’s multiple comparisons test (**A–E**) (*n* = 3 biologically independent samples). *P < 0.05; **P < 0.01; ***P < 0.001; ns, no significant difference.

The contributions of the S protein and its S1 and S2 subunits to IBV entry efficiency were further examined. Using rCEA as a control, the attachment and internalization assays were performed for rLDT, rCEA-LS, -LS1, and -LS2 in both CEK and DF-1 cells. The results of the attachment assay showed that, in both types of cells, rCEA demonstrated significantly higher viral RNA copies than those of rLDT, rCEA-LS, and -LS1, while that of rCEA-LS2 showed no significant difference compared to rCEA (Fig. 4A). Meanwhile, the results of the internalization assay demonstrated that, similar to rLDT, rCEA-LS, -LS1, and -LS2 exhibited internalization capacity comparable to that of rCEA in both CEK and DF-1 cells (Fig. 4B). These findings indicated that the substitution of the S1 subunit, rather than the S2 subunit, significantly reduced the attachment capacity of rCEA, while having no significant impact on viral internalization.

Having established that the residue at position 413 in the S1 subunit played an important role in viral cell tropism, we next evaluated the impact of this site on viral entry. Plasmids encoding trimerized recombinant wild-type S1 proteins of rCEA and rLDT with a C-terminal Strep tag, as well as mutants containing the substitution at position 413, were constructed (Fig. S3A). After transfection into Expi293F cells, the four recombinant proteins, designated Pro-CS1, Pro-CS1(H413Q), Pro-LS1, and Pro-LS1(Q413H), were purified using Strep-Tactin resin and confirmed by Coomassie Brilliant Blue staining and Western blotting (Fig. S3B). These proteins were next incubated with both CEK and DF-1 cells at 4°C for attachment. Cell surface-bound proteins were detected using an antibody against the Strep tag. As shown in Fig. 4C, the capacity of the S1 protein of rCEA (Pro-CS1) to bind both CEK and DF-1 cells was superior to that of the S1 protein of rLDT (Pro-LS1). Compared with that of Pro-CS1, the attachment capacity of the rCEA S1 protein bearing the His413Gln substitution (Pro-CS1-H413Q) was markedly reduced to a level comparable to that of Pro-LS1. Conversely, the Gln413His substitution in the S1 protein of rLDT (Pro-LS1-Q413H) led to an enhancement of attachment capacity.

To further validate this finding, attachment and internalization assays were performed with rCEA-H413Q, which carried the substitution at position 413 in the S1 subunit, and with rCEA and rLDT as controls. In accordance with the attachment characteristics of the parental CEA P23 and LDT3/03 P3, the attachment capacity of rCEA in both CEK and DF-1 cells was significantly higher than that of rLDT. Notably, His413Gln substitution in the S1 protein resulted in a reduction of attachment capacity of rCEA to a level comparable to that of rLDT (Fig. 4D), while this substitution had no detectable effect on viral internalization (Fig. 4E). Subsequently, the attachment capacities of rCEA, rCEA-H413Q, and rLDT in both CEK and DF-1 cells were validated by confocal fluorescence microscopy using viruses labeled with R18, a lipophilic fluorescent dye. As demonstrated in Fig. 4F, both CEK and DF-1 cells inoculated with rCEA exhibited pronounced red fluorescence on the cell surface, whereas cells inoculated with rLDT displayed only minimal fluorescence signals. Compared with rCEA, rCEA-H413Q showed a significant reduction in attachment capacity. These results collectively reveal that residue 413 in the S1 subunit contributes to the expanded cell tropism of CEA by promoting enhanced viral attachment.

### His413 in the S1 subunit mediates efficient binding of IBV to α-2,3-linked sialic acids

**Fig 5:**
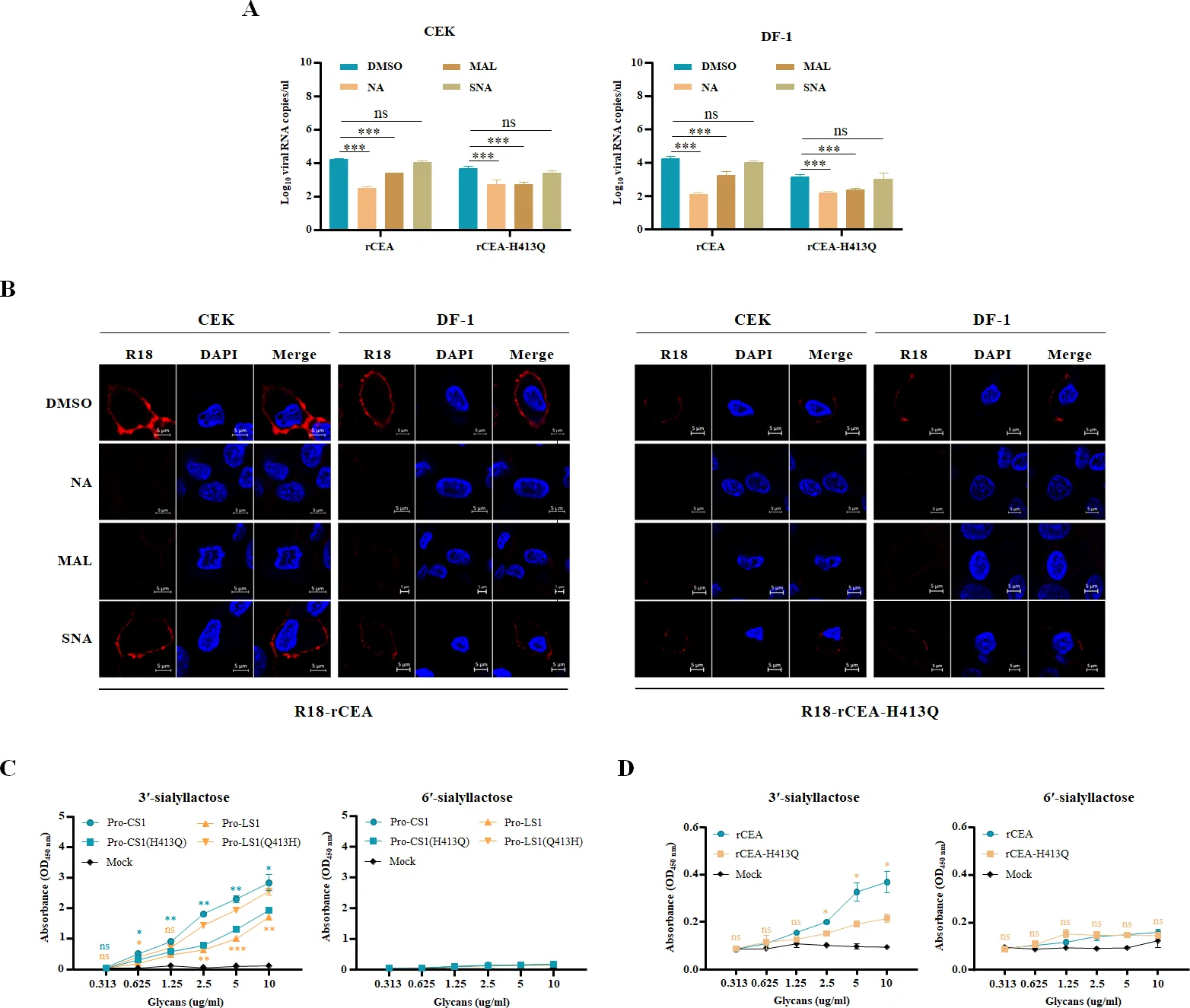
His413 within the S1 subunit is responsible for IBV binding to α-2,3-linked sialic acids. (**A and B**) Evaluation of the effects of α-2,3- and α-2,6-linked sialic acids on attachment of rCEA and rCEA-H413Q to cells. Both CEK and DF-1 cells were pre-treated with NA (to remove cell surface sialic acids), MAL (which specifically binds α-2,3-linked sialic acids), SNA (which specifically binds α-2,6-linked sialic acids), and DMSO (control). Following treatments, cells were subjected to attachment assays with rCEA and rCEA-H413Q. RNA from attached viruses was extracted and quantified by real-time RT-PCR (**A**). Alternatively, pre-treated CEK and DF-1 cells were subjected to attachment assays with R18-labeled rCEA and rCEA-H413Q. Attached virions on the cell surface were visualized by confocal microscopy. Scale bars, 5 μm (**B**). (**C and D**) Assessment of binding properties of rCEA and rCEA-H413Q to α-2,3-linked and α-2,6-linked sialic acid via a solid-phase binding assay. Serially diluted biotinylated 3′-sialyllactose (α-2,3-linked sialic acid analog) and 6′-sialyllactose (α-2,6-linked sialic acid analog) were immobilized on streptavidin-coated plates and then incubated with either S1 proteins [Pro-CS1, Pro-CS1(H413Q), Pro-LS1, and Pro-LS1(Q413H)] or viruses (rCEA and rCEA-H413Q). Elution buffer and allantoic fluids from uninfected SPF eggs served as negative controls for S1 protein and viruses, respectively. Bound S1 proteins were detected using an HRP-conjugated anti-Strep antibody (**C**). Bound viruses were detected using the chicken anti-CEA P20 serum, followed by an HRP-conjugated rabbit anti-chicken secondary antibody (**D**). Colorimetric signals were developed with TMB substrate, and absorbance was measured at 450 nm after stopping the reaction with 0.5 M H₂SO₄. Data were presented as means ± SD, and statistical significance was determined using two-way ANOVA with Sidak’s multiple comparisons test (**A, C, and D**) (*n* = 3 biologically independent samples). *P < 0.05; **P < 0.01; ***P < 0.001; ns, no significant difference.

Although the specific proteinaceous receptors for IBV remain unidentified, α-2,3- and α-2,6-linked sialic acids on the cell surface are recognized as critical attachment factors utilized by IBV to establish infection (27–29). In the present study, the contribution of sialic acids to the attachment of IBV was investigated. Specifically, both CEK and DF-1 cells were pre-treated with *Vibrio cholera* neuraminidase (NA) to remove sialic acids from the cell surface. Furthermore, to determine whether specific sialic acid linkage is required for IBV attachment, both CEK and DF-1 cells were pre-incubated with *Maackia amurensis* lectin (MAL) and *Sambucus nigra* lectin (SNA), which preferentially bind to α-2,3- and α-2,6-linked sialic acids, respectively. Using Newcastle disease virus (NDV) as a model, the effectiveness of the aforementioned reagent in removing or blocking sialic acids was verified (Fig. S4A). Next, rCEA and rCEA-H413Q were attached to both pretreated CEK and DF-1 cells, respectively, and viral RNAs of attached viruses were determined. As shown in Fig. 5A, NA treatment significantly reduced the attachment level of rCEA in both CEK and DF-1 cells. Besides, the pre-incubation of both types of cells with MAL rather than SNA significantly inhibited attachment of rCEA. For rCEA-H413Q, although its intrinsic attachment capacity was lower than that of rCEA, treatment with NA and MAL also effectively reduced its attachment level in both CEK and DF-1 cells. Then the implications of sialic acids in IBV attachment were further visualized by confocal fluorescence microscopy with R18-labeled rCEA and rCEA-H413Q. Pretreatment of both CEK and DF-1 cells with NA and MAL significantly reduced the fluorescent signals of rCEA and rCEA-H413Q attached to the cell surface, whereas SNA treatment exhibited only minimal effects on attachment of rCEA and rCEA-H413Q (Fig. 5B).

Subsequently, biotinylated 3′-sialyllactose and 6′-sialyllactose, α-2,3- and α-2,6-linked sialic acid analogs, were used to assess the glycan-binding properties of viruses. The effectiveness of these two sialyllactose compounds was validated using NDV as a positive control (Fig. S4B). First, the binding activities of recombinant S1 proteins, Pro-CS1 and Pro-CS1(H413Q) derived from rCEA, along with Pro-LS1 and Pro-LS1(Q413H) derived from rLDT, were examined. As shown in Fig. 5C, both Pro-CS1 and Pro-LS1 bound exclusively to 3′-sialyllactose, with no detectable binding to 6′-sialyllactose. Comparatively, Pro-CS1 exhibited significantly stronger binding affinity to 3′-sialyllactose compared with Pro-LS1. Notably, His413Gln substitution in the rCEA S1 reduced binding affinity to 3′-sialyllactose, whereas the reciprocal substitution Gln413His in the rLDT S1 enhanced it. None of the substitutions significantly altered the binding affinity of the S1 protein to 6′-sialyllactose. To validate these findings, the glycan-binding assay was performed with two viruses, rCEA and rCEA-H413Q. The results demonstrated that both rCEA and rCEA-H413Q exhibited preferential binding to 3′-sialyllactose, rather than 6′-sialyllactose, and the affinity of rCEA was higher than that of rCEA-H413Q (Fig. 5D). These findings indicate that the histidine residue at position 413 within the S1 subunit of IBV enhances viral attachment by specifically increasing the viral binding to α-2,3-linked sialic acids.

### Gangliosides are involved in IBV entry via caveolae-mediated endocytosis

**Fig 6:**
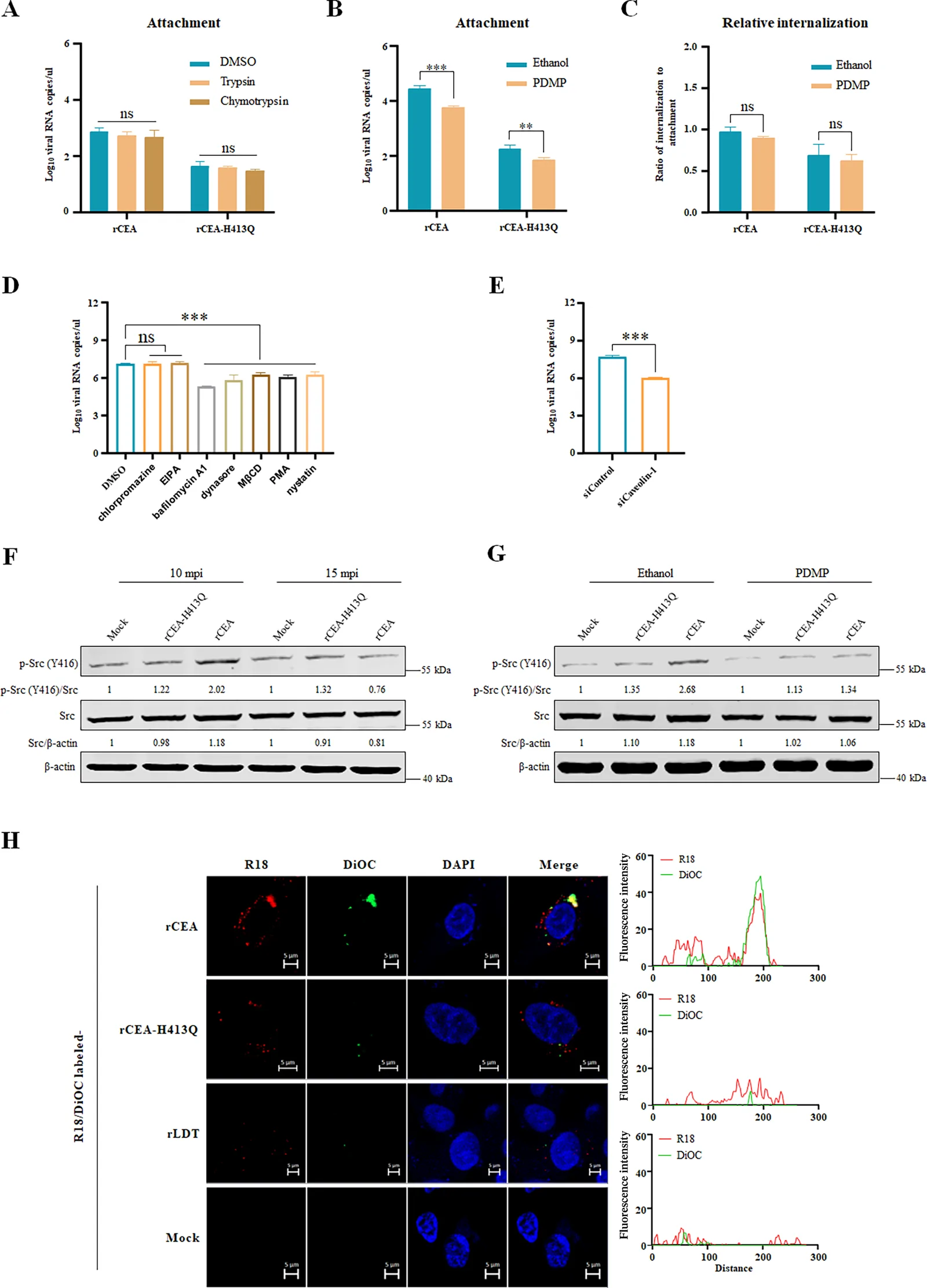
IBV enters cells via the CavME by binding to gangliosides through His413 in the S1 subunit. (**A–C**) Evaluation of the effects of glycoproteins and gangliosides on viral attachment and internalization. DF-1 cells were pre-treated with trypsin or chymotrypsin to remove surface glycoproteins, followed by attachment assays with rCEA and rCEA-H413Q, with DMSO as a control. RNA from attached viruses was extracted and quantified by real-time RT-PCR (**A**). Alternatively, DF-1 cells were pre-treated with PDMP to reduce cell surface glycosphingolipids, with ethanol as a control. Then the attachment (**B**) and internalization (**C**) assays were performed with rCEA and rCEA-H413Q. Attached and internalized viral RNAs were extracted and quantified by real-time RT-PCR, with internalization efficiency calculated as the ratio of internalized to attached RNA. (**D**) Effects of pharmacological inhibitors of endocytic pathways on IBV replication. DF-1 cells were pre-treated with DMSO, bafilomycin A1 (endosome acidification inhibitor), dynasore (dynamin inhibitor), MβCD, PMA, nystatin (CavME inhibitors), chlorpromazine (CME inhibitor), or EIPA (macropinocytosis inhibitor) and then inoculated with rCEA in the presence of inhibitors. Viral RNA in culture supernatants was quantified at 24 hpi. (**E**) Effect of caveolin-1 knockdown on IBV internalization. DF-1 cells transfected with caveolin-1-specific siRNA (siCaveolin-1) or control siRNA (siControl) were subjected to an internalization assay with rCEA. Internalization efficiency was calculated as described in panel **C**. (**F**) Determination of levels of Src Y416 phosphorylation during IBV entry. DF-1 cells were inoculated with rCEA and rCEA-H413Q at 4°C for 1 h. After shifting to 37°C, the cells were harvested at 10 and 15 mpi. The levels of p-Src (Y416), total Src, and β-actin in cell lysates were detected by Western blotting. The ratios of p-Src (Y416)/Src and Src/β-actin were shown. (**G**) Examination of the effect of gangliosides on virus-induced Src phosphorylation. DF-1 cells were pre-treated with PDMP or ethanol. The inoculation with rCEA and rCEA-H413Q and detection of indicated proteins were performed as described in panel **F**. (**H**) Visualization of IBV fusion with intracellular endosomes. DF-1 cells were inoculated with R18/DiOC double-labeled rCEA, rCEA-H413Q, and rLDT and subjected to internalization assays. Red (R18) and green (DiOC) fluorescence were visualized by confocal microscopy (left). Scale bars, 5 μm. Colocalization of R18 and DiOC signals was analyzed using ImageJ software (right). Data were presented as means ± SD, and statistical significance was determined using two-way ANOVA with Sidak’s multiple comparisons test (**A–C**), one-way ANOVA with Bonferroni’s multiple comparisons test (**D**), or unpaired two-tailed Student’s *t*-test (**E**) (*n* = 3 biologically independent samples). **P < 0.01; ***P < 0.001; ns, no significant difference.

Sialic acids are predominantly located at the terminal positions of glycoconjugates, including glycoproteins and gangliosides (43). In this study, we examined whether sialic acid-containing glycoproteins or gangliosides are preferentially involved in the entry process of IBV. To differentiate the potential effects of glycoproteins and glycolipids on viral attachment, chymotrypsin and trypsin, two proteases that cleave peptide bonds, and PDMP (N-[(1R,2R)-2-hydroxy-1-(4-morpholinylmethyl)-2-phenylethyl]-decanamide, monohydrochloride), an inhibitor that reduces the level of glycosphingolipids on the cell surface, were adopted (44, 45). Since CEK cells are relatively sensitive to proteases and PDMP treatment, the evaluation of their effects on IBV entry was performed in DF-1 cells. The results demonstrated that treatment with chymotrypsin and trypsin did not affect the attachment of rCEA and rCEA-H413Q to DF-1 cells, whereas PDMP treatment significantly reduced the attachment of these two viruses (Fig. 6A and B). In contrast, internalization of both viruses was unaffected by proteases or PDMP treatment (Fig. 6C). These results suggest that sialic acid-containing gangliosides, rather than glycoproteins, are associated with IBV entry into cells.

The cell entry mechanism of IBV remains poorly characterized; however, several studies have demonstrated that IBV is capable of hijacking multiple cellular endocytic pathways for entry, including CME and CavME (18, 32). Recently, we revealed that gangliosides could mediate the entry of NDV into cells by triggering the CavME (46). To investigate the specific pathways exploited by IBV for entry, pharmacological inhibitors were utilized to block various endocytic pathways, and their effects on rCEA replication were determined. As shown in Fig. 6D, pretreatment with endosome acidification inhibitor bafilomycin A1, dynamin inhibitor dynasore, as well as three inhibitors of CavME, namely methyl-β-cyclodextrin (MβCD), phorbol 12-myristate 13-acetate (PMA), and nystatin, resulted in a significant decrease in the replication level of rCEA in DF-1 cells, while CME inhibitor chlorpromazine and macropinocytosis inhibitor 5-(N-methyl-N-isobutyl)­amiloride (EIPA) had no effect on the replication of rCEA. Based on the targets of these endocytic pathway inhibitors, we hypothesized that the CavME is involved in the entry of rCEA. To address the potential off-target effects inherent to pharmacological inhibitors, we employed specific siRNA designed in our previous work to knock down the expression of caveolin-1, a vital factor in CavME, thereby validating this hypothesis (47, 48). DF-1 cells were transfected with siRNA targeting caveolin-1 (siCaveolin-1) or a control siRNA (siControl) and then inoculated with rCEA to conduct the internalization assay. As shown in Fig. 6E, a significant reduction in viral RNA copies was observed in caveolin-1 knockdown cells compared with that in siControl-transfected cells, indicating that the entry of rCEA was impaired. These results further indicate that rCEA can enter DF-1 cells via the CavME.

Activation of the non-receptor tyrosine kinase Src via the autophosphorylation of tyrosine at 416 (Y416) is a hallmark of CavME (49). Thus, the level of Y416 phosphorylation of Src in DF-1 cells during the early entry stage of rCEA and rCEA-H413Q infection was examined via Western blotting. As shown in Fig. 6F, both rCEA and rCEA-H413Q could induce an increased level of Src Y416 phosphorylation at 10 min post-inoculation (mpi), with rCEA eliciting a more pronounced increase. At 15 mpi, rCEA-induced phosphorylation of Src Y416 was decreased, whereas rCEA-H413Q still induced a slight increase in Src Y416 phosphorylation, indicating that rCEA induced more rapid and robust phosphorylation of Src Y416. Next, we examined whether Src Y416 phosphorylation induced by IBV required gangliosides. After PDMP treatment to reduce cell surface gangliosides, the cells were assessed at 10 mpi for changes in Y416 phosphorylation of Src induced by rCEA and rCEA-H413Q. The results showed that PDMP treatment significantly reduced the level of Src Y416 phosphorylation induced by both viruses, especially rCEA (Fig. 6G). This indicated that Src Y416 phosphorylation induced by IBV was ganglioside-dependent.

Enveloped viruses that enter cells via endocytosis accomplish fusion in the intracellular endosomes. To further examine the fusion of IBV virions with the intracellular endosomes following the CavME, rCEA, rCEA-H413Q, and rLDT were labeled with two lipophilic fluorescent dyes, DiOC (green) and R18 (red), as previously described (32, 50), and then subjected to an internalization assay. Due to the quenching effect of R18, the green fluorescence of DiOC can be observed only after the fusion of the viral envelope with the cellular membrane, which dilutes the R18. The confocal fluorescence microscopy demonstrated that marked red fluorescence of R18 and green fluorescence of DiOC were observed exclusively in the cytoplasmic region of rCEA-inoculated cells, and these two fluorescence signals exhibited clear colocalization. In contrast, only sporadic fluorescence signals were observed in rCEA-H413Q-inoculated cells, and both fluorescence signals were almost invisible in rLDT-inoculated cells (Fig. 6H). These results indicated that His413 in the S1 subunit was required for the efficient entry of IBV. Taken together, these findings demonstrate the role of gangliosides in the entry of IBV into cells via CavME, with His413 in the S1 subunit critical for initiation of CavME and fusion of IBV with intracellular endosomes.

## DISCUSSION

Since its initial discovery in the 1930s, IBV has emerged as one of the most significant threats to the global poultry industry. The scarcity of IBV strains adapted to cell culture, and especially the limited efficiency of reverse genetics systems for virus rescue, has long been bottlenecks hindering investigation of the molecular basis of IBV pathogenicity and the development of novel vaccines. Assembly of full-length cDNA poses the greatest challenge in the reverse genetics system of CoVs due to the large viral genome. Recently, a bacteria-free CPER method, initially developed for the construction of infectious clones of flaviviruses (38), has been successfully applied to the rescue of SARS-CoV-2 (39, 40). In addition to its application in these positive-sense RNA viruses, this technique has also been successfully utilized in the development of reverse genetics systems for rabies virus, which possesses a negative-sense RNA genome (51). These findings establish CPER as a versatile platform for genetic manipulation of RNA viruses. Compared with previously reported reverse genetic platforms for IBV, the CPER system offers several advantages, including simplified procedures, low technical requirements, independence from specific restriction enzyme sites, and the elimination of complex cloning steps in bacterial or yeast hosts. In this study, utilizing LDT3/03 and its CEF cell-adapted CEA strain (9), a reverse genetics platform of IBV based on the CPER method was established for the first time. The recovered viruses retained the cell infection characteristics of their respective parental viruses. Subsequently, a series of chimeric or single-site substituted viruses were rescued for elucidation of the molecular mechanism underlying adaptation of the CEA strain to chicken-derived fibroblast cells. The expanded applicability of this strategy to IBV demonstrated in this study further indicates that this system is a powerful tool for advancing the basic and applied research on IBV as well as other CoVs.

CoV S protein, a class I fusion protein anchored in the viral envelope, is the main determinant of viral entry and cellular tropism. Two cleavage sites, S1/S2 and S2′, which are located at the S1/S2 boundary and immediately upstream of the putative fusion peptide within the S2 subunit, respectively, have been identified in the CoVs S protein. The cleavage at the S1/S2 site can occur at different stages of the viral life cycle, depending on the specific amino acid motif present in certain CoVs (52). However, accumulating evidence suggests that cleavage at the S1/S2 site may not be obligatory for all CoVs to establish infection (18, 53, 54). This viewpoint also applies to IBV, as all IBV isolates possess the S1/S2 cleavage site that can be processed by furin protease during biosynthesis, whereas almost all of them are incapable of infecting cells cultured *in vitro*. In contrast, the cleavage at the S2′ site has been considered a critical event for entry of CoVs, as it exposes an internal fusion peptide within the S2 subunit for the initiation of fusion. The conserved XXXR/S motif at the CoVs S2′ site determines the associated protease responsible for cleavage at this site and the specific location where membrane fusion occurs (plasma membrane and/or endosome), with the first three amino acids playing a critical role in this process (18, 52, 55–57). For instance, SARS-CoV-2 can utilize transmembrane protease, serine 2 (TMPRSS2) or cathepsin L to cleave the S protein at the S2′ site, thereby initiating fusion either at the cell surface or within endosomal compartments (57). For IBV, it is only known that the unique furin cleavage motif (RRKR/S) at the S2′ site is responsible for the extended cell tropism of the Beaudette strain (13). Binding mediated by this S2′ site to the attachment receptor HS, along with cleavage by furin protease, facilitates the cellular entry of Beaudette strain (17, 18). Further studies have shown that Beaudette strain enters various cells via the endocytic pathways and initiates membrane fusion in late endosome–lysosome (32, 58). In the present study, we demonstrated that the CEA strain also enters cells via endocytosis, and membrane fusion of virions with host cells occurs intracellularly rather than at the plasma membrane. Thus, unlike other CoVs that can enter cells via both fusion at the plasma membrane and receptor-mediated endocytosis, endocytic entry appears to be the common strategy employed by IBV to enter target cells. It is noteworthy that the CEA strain lacks the furin cleavage motif at the S2′ site, which suggests that this strain may employ distinct mechanisms for S protein activation and membrane fusion initiation compared with the Beaudette strain. This mechanism may represent a common strategy for IBV entry, as nearly all IBV isolates, similar to CEA strain, lack a furin cleavage motif at the S2′ site. Additionally, IBV strains containing different motifs at the S2′ site likely utilize distinct conditions within endosomal compartments (e.g., pH, proteases, ions, and potential intracellular receptors) to cleave and activate the S protein, thereby initiating membrane fusion. This requires further elucidation in future studies.

The S1 subunit of CoVs generally comprises two domains, the NTD and CTD, with the CTD exhibiting greater diversity, displaying low or no structural similarity across different genera (21). Among the four genera of CoVs, the S1 CTD of IBV exhibits notable structural differences from those of the other three genera, characterized by a weakened β-sandwich core structure and two extended loops on the edge of the core structure. In contrast to the S1 NTD, for which the specific RBD has been identified, although studies have indicated that the IBV S1 CTD can function as an RBD recognizing unknown receptors on chicken cells, the precise region of the RBD is yet to be determined (30). Additionally, the unique structural features of the IBV S1 CTD suggest that it may recognize receptor types different from those recognized by the other three genera. In the present study, we identified that the amino acid at position 413 within the S1 CTD of IBV is a key determinant influencing viral entry by mediating the viral binding to α-2,3-linked sialic acids. This finding indicates that, in addition to its hypothetical role in binding proteinaceous receptors similar to CTDs of other CoVs, the S1 CTD of IBV is also capable of interacting with carbohydrates to facilitate infection, thereby expanding the current understanding of IBV receptor recognition. The structure determination of the S protein of the CEA strain remains unresolved. According to the cryo-EM structure of the S protein of IBV M41 strain, the amino acid at position 413 of the CEA S1 subunit is predicted to be located within the β-sheet layers of the β-sandwich core structure. On this basis, we propose the hypothesis that the histidine residue at position 413 in CEA S1 may be prone to induce conformational changes in the core structure, facilitating a transition from a “lying” to a “standing” state. This transition could provide a structural basis for efficient binding of the S1 CTD to sialic acids (30, 59). Additionally, the Gln413His substitution that occurred during the passage of the LDT3/03 strain to CEF cells may enhance the ability to form hydrogen bonds with the sugar ligands, thereby promoting the binding of the S1 CTD to sialic acids, as reported in SARS-CoV-2 (60, 61).

Although the binding of the CoV S1 NTD to carbohydrates is considered a low-affinity, non-specific interaction, the sialic acid-binding mediated by this domain may still facilitate viral adaptation to new host environments, thereby expanding host cell tropism (21). In this study, we showed that a specific amino acid in the S1 CTD can also enhance the binding of IBV to sialic acids linked to gangliosides, thereby facilitating viral attachment and initiating infections. For most CoVs, engagement with proteinaceous receptors triggers conformational changes in the S protein, leading to dissociation of S1 from S2 and exposure of the S2′ cleavage site for protease processing, thereby initiating membrane fusion (62–64). As an attachment factor, sialic acids participate in the infection process of CoVs among different genera. However, the weak binding to sialic acids does not trigger a conformational change in the S protein, and the specific contribution of sialic acids in CoV entry remains insufficiently elucidated (65). In fact, it appears that most CoVs utilize endocytosis to deliver their genetic material into the cytoplasm, thereby initiating productive infection. The most commonly employed and mechanistically best-characterized endocytic pathway among CoVs is CME, the initiation of which typically depends on the binding of viruses to transmembrane protein receptors. For instance, both SARS-CoV and SARS-CoV-2 recruit clathrin and a series of accessory factors to initiate CME through binding to the transmembrane protein ACE2 (66–69). Similarly, the IBV Beaudette strain is also capable of entering various cell types via the CME pathway; however, the transmembrane protein receptors or cofactors mediating CME initiation by IBV remain to be identified (66–69). Herein, we showed that sialic acid-containing gangliosides, rather than proteinaceous receptors, were involved in the entry of IBV CEA strain into cells via the CavME pathway. We speculated that CEA strain may act as a multivalent ligand to crosslink gangliosides within caveolae via binding to terminal sialic acids, thereby activating Src to initiate the CavME pathway (46, 70, 71). This is consistent with the strategy of CoVs cell tropism expansion, whereby the virus first enhances its attachment by binding to carbohydrate molecules on the cell surface, thereby facilitating infection (21). Interestingly, in addition to entering human H1299 cells via the CME pathway, the Beaudette strain can also utilize the CavME pathway to enter the same cell type (32). We infer that differences among IBV strains in S protein sequences, which affect receptor binding and proteolytic cleavage activation, together with the types and abundances of receptors and accessory factors in cells, collectively determine the cell tropism of IBV and its pathway of cell entry. Furthermore, whether proteinaceous receptors are additionally involved in the viral entry process following the binding of IBV to terminal sialic acids on gangliosides, and which specific ganglioside subtypes mediate IBV invasion, remain to be further explored.

During the adaptation of CoVs to new environments as well as new hosts, in addition to adaptive amino acid substitutions in the S protein that alter receptor binding and cell/tissue tropism, accumulative substitutions in other structural and nonstructural proteins can also affect various stages of the viral life cycle, including RNA synthesis and assembly of double-membrane vesicles, thereby ensuring productive viral infection (72–75). For IBV, it has been found that specific amino acid substitutions in nsp9, nsp13, and nsp15 enhance viral replication by promoting subgenomic RNA synthesis and immune modulation (76–78). In this study, we also constructed chimeric viruses in which the S1 and S2 subunits, and the whole S gene of LDT3/03 P3 were replaced with the corresponding regions of CEA P23, designated rLDT-CS, rLDT-CS1, and rLDT-CS2, respectively. The S gene sequences of rLDT-CS, -CS1, and -CS2 were confirmed by sequencing (Fig. S5A). Among these viruses, both rLDT-CS and rLDT-CS1 exhibited attachment capability similar to that of rCEA, whereas the attachment capability of rLDT-CS2 remained comparable to that of rLDT (Fig. S5B). As expected, no significant differences in viral internalization efficiency were observed among all viruses (Fig. S5C). It is noteworthy that rLDT-CS, rLDT-CS1, and rLDT-CS2 each exhibited only limited proliferation in DF-1 cells, which was slightly higher than that of rLDT but significantly lower than that of rCEA (Fig. S5D). Besides the amino acid differences in the S protein, multiple amino acid substitutions were also observed between the CEA P23 and the LDT3/03 P3 in other structural and non-structural proteins (Fig. 1C). A recent study has revealed that mutations in nsp4, E protein, and S2 subunit collectively determine the expanded mammalian cell tropism of IBV (79). Therefore, although the present study demonstrates that specific amino acid substitution in the S1 CTD can facilitate IBV infection by promoting enhanced attachment, other structural and non-structural proteins also play indispensable roles in the cellular adaptability of IBV. Whether the mutations in these proteins contribute to the expansion of IBV cell tropism through mechanisms, such as modulating viral RNA synthesis, facilitating intercellular viral spread, and counteracting host innate immune responses, warrants further investigation. In summary, the S1-mediated enhancement of attachment capacity should be the prerequisite for the expansion of IBV cell tropism, while the synergistic effects provided by other viral proteins further ensure efficient viral replication in host cells. Furthermore, a 197-nt deletion was found in the putative ORF7 region downstream of the N gene in the CEA P20 genome (9). It is reasonable to assume that this 197-nt deletion does not affect viral RNA replication, as it does not disrupt the formation of the hairpin-type pseudoknot structure within the 3′ UTR. This inference is supported by the observation that recombinant viruses containing ORF7 of varying lengths, constructed using the Beaudette strain backbone, exhibit comparable growth kinetics *in vitro* (80). However, it remains to be further verified whether the potential protein encoded by ORF7 is detrimental to viral replication in cells and thus is deleted during IBV adaptation to cells.

This study established, for the first time, a reverse genetics platform for IBV based on CPER, thereby providing a powerful tool for research on IBV biology, as well as vaccine and countermeasure development. On this basis, the molecular mechanism by which the specific amino acid substitution in the S1 CTD facilitated viral attachment for productive infection was elucidated. Our findings provide novel insights into the strategy employed by IBV for cell adaptation and expand the understanding of the cellular entry mechanisms of CoVs.

## MATERIALS AND METHODS

### Eggs, cells, and viruses

SPF-embryonated chicken eggs were obtained from the National Poultry Laboratory Animal Resource Center, Harbin Veterinary Research Institute (HVRI), Chinese Academy of Agricultural Sciences (CAAS). Primary CEF and CEK cells were prepared from 9- and 18-day-old SPF-embryonated chicken eggs and cultured as previously described, respectively (3, 81). Immortalized chicken embryonic fibroblast cell line DF-1 was purchased from the American Type Culture Collection (ATCC) and maintained in Dulbecco’s modified Eagle’s medium (DMEM) containing 10% heat-inactivated fetal bovine serum (FBS) at 37°C with 5% CO_2_. Expi293F cells were cultured in Expi293 Expression Medium (Thermo Fisher Scientific, Waltham, MA, USA) as described previously (82). An embryonated egg-adapted IBV strain, tl/CH/LDT3/03 (LDT3/03), and its CEF-adapted strain, CEA P20, were preserved in our laboratory (9, 83). LDT3/03 and CEA P20 were further propagated for three passages in embryonated eggs and CEF cells, respectively, yielding viral stocks designated LDT3/03 P3 and CEA P23 that were used in this study.

### Plasmid construction

UTR linker fragments of CEA P23 and LDT3/03 P3, which contain the genomic 3′ terminal 320 nt of CEA P23 or 3′ terminal 516 nt of LDT3/03 P3, HDVr sequence, SV40 poly A signal, CMV promoter sequence, and genomic 5′ terminal 529 nt of CEA P23 or LDT3/03 P3, were synthesized and cloned into the pUC19 vector. The resulting plasmids were designated pUTR-CEA and pUTR-LDT3/03, respectively.

Construction of the plasmids containing the genomic fragments of CEA P23 and LDT3/03 P3 was performed as previously described (40). Briefly, first-strand cDNAs of CEA P23 and LDT3/03 P3 were synthesized from extracted viral RNAs using a PrimeScript II 1st Strand cDNA Synthesis Kit (Takara) with random hexamer primers. Then nine overlapping genomic fragments (G1–G9) covering the entire genome of CEA P23 and LDT3/03 P3 were amplified by KOD-Plus-Neo polymerase (Toyobo, Shanghai, China) with synthesized cDNAs and cloned into the pMD18-T vector (Takara). The resultant plasmids were designated pCEA-G1–G9 and pLDT-G1–G9, respectively. To distinguish between the recovered viruses and the original viruses, silent mutations T2173C and T14382A were introduced into pCEA-G1 and pLDT-G6 as genetic markers, respectively, via site-directed mutagenesis. In addition, CDS regions of the S gene, S1, and S2 subunits in plasmid pCEA-G8 were replaced by those in pLDT-G8 through overlap PCR, as previously described (84). The resulting plasmids were designated pCEA-G8-LS, -LS1, and -LS2, respectively. Furthermore, A599G, C1239A, and T1456C mutations within the S1 CDS of pCEA-G8, which result in Asn200Ser, His413Gln, and Phe486Leu amino acid substitutions in the S1 subunit, were introduced via site-directed mutagenesis, respectively. The resultant plasmids were designated pCEA-G8-N200S, -H413Q, and -F486L, respectively.

To construct the helper plasmid expressing the N protein, the CDS of the CEA P23 N gene was amplified from viral cDNA and cloned into the pCAGGS vector (85). The resultant plasmid was named pCAG-N. To construct plasmids expressing CEA P23 S1 subunit, the S1 CDSs were amplified from pCEA-G8 and pCEA-G8-H413Q using primers with appended sequences encoding a C-terminal fibritin trimerization domain, a Strep tag, and a Flag tag. The amplified fragments were then cloned into the pCAGGS vector, yielding pCAG-CS1 and pCAG-CS1-H413Q, respectively. For construction of LDT3/03 P3 S1 subunit expressing plasmid, the S1 CDS was amplified from pLDT-G8 and cloned into the pCAGGS vector, yielding pCAG-LS1. Subsequently, an A1239C mutation in the S1 CDS of pCAG-LS1, which results in a Gln413His substitution in the S1 subunit, was performed via site-directed mutagenesis. The resultant plasmid was designated pCAG-LS1-Q413H. Sequences of all constructed plasmids were confirmed by Sanger sequencing (BGI, Beijing, China). The details of primers used for plasmid construction and mutagenesis are provided in Table S1.

### Construction of infectious clone via CPER

The infectious clone of IBV was assembled by CPER as previously described (39, 40). Briefly, for the CEA P23 strain, nine cDNA fragments (F1–F9) and a UTR linker, each with complementary ends sharing approximately 50-nucleotide overlaps, were amplified from plasmids pCEA-G1–G9 and pUTR-CEA, respectively, using specific primers (Table S1). Equimolar amounts (0.1 pmol each) of the 10 resulting DNA fragments were used as templates for CPER in a 50 μL reaction volume with PrimeSTAR GXL DNA Polymerase (Takara). The cycling conditions of CPER were as follows: an initial denaturation at 98°C for 2 min, followed by 35 cycles of denaturation at 98°C for 10 s, annealing at 55°C for 15 s, extension at 68°C for 15 min, and a final extension at 68°C for 15 min. Following the aforementioned procedure, the infectious clone of the LDT3/03 P3 strain was assembled using DNA fragments amplified from pLDT-G1–G9 and pUTR-LDT3/03. To construct chimeric infectious clones of CEA P23 carrying the S, S1, or S2 CDSs from LDT3/03 P3, as well as site-specific mutations, the corresponding chimeric genomic fragments were generated by replacing pCEA-G8 with pCEA-G8-LS, -LS1, -LS2, -N200S, -H413Q, and -F486L, respectively. Subsequently, CPER was performed using the above-described method. The resultant CPER products were designated CEA-FL, LDT-FL, CEA-FL-LS, -LS1, -LS2, -N200S, -H413Q, and -F486L, respectively.

### Virus recovery

For recovery of the infectious viruses, 25 μL of CPER products without purification were co-transfected with 2.5 μg helper plasmid pCAG-N into DF-1 cells seeded in 6-well plates using *Trans* IT LT-1 (Mirus, Madison, WI, USA), according to the manufacturer’s instructions. At 12 h post-transfection, the culture supernatants of transfected cells were replaced with DMEM containing 5% FBS. After 3 days, the cells and culture supernatants were harvested and subjected to one freeze-thaw cycle before being inoculated into the allantoic cavities of 9-day-old SPF embryonated chicken eggs. At 72 hpi, allantoic fluids were collected and further amplified by inoculating into 9-day-old SPF eggs once to generate viral stocks. The presence of recovered viruses in allantoic fluids was examined via RT-PCR, Western blotting, and electron microscopy as described previously (34, 83). The recovered viruses derived from CPER products CEA-FL, LDT-FL, CEA-FL-LS, -LS1, -LS2, -N200S, -H413Q, and -F486L were named rCEA, rLDT, rCEA-LS, -LS1, -LS2, -N200S, -H413Q, and -F486L, respectively.

### Treatment of cells with reagents and siRNA

To assess the role of sialic acids in viral attachment, CEK and DF-1 cells cultured in 6-well plates or confocal dishes were pre-treated with 60 mU *Vibrio cholera* NA (Roche, Switzerland), 500 µg/mL MAL, or 500 µg/mL SNA (Vector Labs, USA) for 1 h at 37°C, and DMSO was used as a control (46). To evaluate the contribution of glycoproteins to viral attachment, DF-1 cells in 6-well plates were incubated with 10 µM trypsin or chymotrypsin (Sigma, USA) for 30 min at 37°C, with DMSO as the control. To investigate the role of gangliosides in viral attachment, internalization, or Src activation following viral entry, DF-1 cells in 6-well plates were pre-treated with 5 µM PDMP (Cayman, USA) in DMEM containing 2% FBS for 72 h at 37°C, with ethanol as the control. After the respective treatments, all cells were then subjected to attachment, internalization, or infection assays.

To evaluate the involvement of caveolin-1 in viral internalization, the siRNA targeting chicken caveolin-1 (siCaveolin-1: 5′-CCGAGCCACAGUACGGAAA-3′) and a negative control siRNA (siControl) with no specific target in chicken cells were used as previously described (47, 48). DF-1 cells were transfected with 100 nM siRNA using the Lipofectamine RNAiMAX Reagent (Thermo Fisher Scientific) according to the manufacturer’s instructions. At 36 h post-transfection, cells were subjected to internalization assays.

To investigate the entry pathways utilized by IBV, DF-1 cells in 6-well plates were pre-treated with various pharmacological inhibitors targeting distinct endocytic pathways for 1 h at 37°C. These included bafilomycin A1 (an inhibitor of endosomal acidification), dynasore (a dynamin inhibitor), three inhibitors of CavME, namely, MβCD, PMA, and nystatin, as well as chlorpromazine (an inhibitor of CME) and 5-(N-methyl-N-isobutyl)­amiloride (EIPA) (an inhibitor of macropinocytosis) (Sigma). DMSO was included as a control. After treatments, all cells were subjected to a viral infection assay. The working concentrations of all reagents were predetermined by cell viability assays using CCK-8 in our previous study (48).

### Attachment and internalization assays

The amount of virus was quantified and standardized by measuring the viral RNA copy numbers as previously described (14–16, 78), as the TCID_50_ assay was not applicable to IBV strains that cannot replicate in cells. For viral attachment assays, CEK, CEF, and DF-1 cells cultured in 6-well plates were inoculated with the indicated IBV strains at a dose of 5 × 10^9^ viral RNA copies and incubated at 4°C for 1 h to allow viral attachment. After washes with cold PBS, the cells were processed for RNA extraction, and viral RNA was quantified by real-time RT-PCR (48, 86). In addition, CEK and DF-1 cells pre-treated with NA, MAL, or SNA, and DF-1 cells pre-treated with trypsin, chymotrypsin, or PDMP were used for attachment assays of the indicated viruses following the same procedure.

For internalization assays, CEK, CEF, and DF-1 cells were inoculated with the indicated IBV strains at a dose of 5 × 10^9^ viral RNA copies for 1 h at 4°C. After washes with cold PBS, the cells were further incubated with pre-warmed DMEM for 1 h at 37°C for viral internalization. Next, cells were treated with citrate buffer (pH 3.0) to remove non-internalized virions (46, 48, 86). Then, RNA from internalized viruses was extracted and quantified by real-time RT-PCR. In addition, DF-1 cells pre-treated with PDMP or transfected with siRNA targeting caveolin-1 were used for the internalization assay of the indicated viruses following the above-described procedure. Internalization efficiency was calculated by normalizing the copy number of internalized viral RNA to that of attached RNA.

### Viral infection assay

To assess viral replication kinetics, CEK, CEF, and DF-1 cells seeded in 12-well plates were inoculated with the indicated IBV strains at a dose equivalent to 5 × 10^7^ viral RNA copies per well. After incubation for 1 h at 37°C, the inoculum was removed, and the cells were washed twice with PBS prior to maintenance in DMEM supplemented with 2% FBS. At indicated time points post-inoculation, culture supernatants were collected for viral RNA extraction and quantification by real-time RT-PCR, and cells were subjected to immunofluorescence assay.

To assess the effects of inhibitors on the replication of IBV, DF-1 cells pre-treated with the indicated inhibitors were inoculated with rCEA at a dose of 5 × 10^7^ viral RNA copies in the presence of the inhibitors for 1 h at 37°C. After incubation, the cells were maintained in DMEM containing the inhibitors and 2% FBS at 37°C. At 24 hpi, culture supernatants were collected for viral RNA extraction and quantification by real-time RT-PCR.

To examine Src activation during viral entry, DF-1 cells either untreated or pre-treated with PDMP were inoculated with the indicated IBV strains at a dose of 1 × 10^10^ viral RNA copies and incubated at 4°C for 1 h to allow attachment. After washing with PBS to remove unbound virus, cells were shifted to 37°C and harvested at 10 or 15 mpi. Cell lysates were subjected to Western blotting.

### S1 protein expression and purification

Expi293F cells were transfected with pCAG-CS1, pCAG-CS1-H413Q, pCAG-LS1, or pCAG-LS1-Q413H using the 293 SFM Kit (Kairui Biotech, Zhuhai, China) according to the manufacturer’s instructions. After 5 days, cell culture supernatants were harvested and incubated overnight at 4°C with Strep-Tactin XT 4Flow resin (IBA Lifesciences, Göttingen, Germany). The resin was then extensively washed with wash buffer (50 mM Tris-HCl, 150 mM NaCl, pH 8.0), and bound proteins were eluted using wash buffer supplemented with 2.5 mM D-biotin. Eluted fractions were concentrated using a 30-kDa cutoff centrifugal filter. The purified proteins, designated Pro-CS1, Pro-CS1(H413Q), Pro-LS1, and Pro-LS1(Q413H), were then resolved by 10% SDS-PAGE and analyzed via Coomassie Brilliant Blue staining (Biodragon, Suzhou, China) and Western blotting.

### RT-PCR and real-time RT-PCR

Viral RNA extracted from allantoic fluids, cell lysates, or culture supernatants was subjected to RT-PCR or real-time RT-PCR. For RT-PCR, fragments covering either the G8 region or the introduced genetic markers were amplified with the PrimeScript One Step RT-PCR Kit Ver.2 (TaKaRa) using corresponding primers (Table S1). The resulting amplicons were analyzed via agarose gel electrophoresis and verified by Sanger sequencing. Real-time RT-PCR was performed as described previously (87). To quantify viral RNA copies, a standard curve was generated using serial 10-fold dilutions of a DNA template containing the target 3′ UTR sequence at known concentrations. Viral RNA copy numbers were calculated based on the standard curve.

### Immunofluorescence assay

Virus-infected cells were washed and fixed with 4% paraformaldehyde. The cells were then incubated with primary antibodies: mouse anti-IBV N monoclonal antibody (4F10) (88) or anti-dsRNA J2 IgG2a monoclonal antibody (English and Scientific Consulting, Szirák, Hungary), followed by FITC-conjugated goat anti-mouse secondary antibody (Sigma). Nuclei were stained with 4′,6′-diamidino-2-phenylindole (DAPI) (Beyotime, Shanghai, China). Fluorescent images were captured using an inverted fluorescence microscope.

### Confocal microscopy

rCEA, rCEA-H413Q, and rLDT were propagated in CEK cells and labeled with either a single lipophilic dye (R18) or a combination of two lipophilic dyes (R18 and DiOC) (Thermo Fisher Scientific) as previously described (32, 86). To visualize viral attachment, CEK and DF-1 cells either untreated or pre-treated with NA, MAL, and SNA were inoculated with R18-labeled viruses as described in the viral attachment assay. After fixation and staining of the nuclei with DAPI, the cells were observed using confocal microscopy. To visualize viral internalization, DF-1 cells were inoculated with R18/DiOC-labeled viruses as described in the viral internalization assay. Then, the cells were fixed, stained with DAPI, and observed using confocal microscopy. All images were acquired using an LSM980 confocal laser-scanning microscope (Zeiss, Oberkochen, Germany).

### Western blotting

Virus-infected cells were harvested and lysed on ice for 30 min using radioimmunoprecipitation assay lysis buffer (Beyotime) supplemented with protease and phosphatase inhibitors. Cell lysates were clarified by centrifugation at 12,000 × *g* for 10 min at 4°C, and protein concentrations of the resulting supernatants were determined using a Bradford assay kit (Beyotime). Equal amounts of protein were separated by 10% SDS-PAGE and transferred onto nitrocellulose membranes. Membranes were blocked with 5% skim milk and then incubated with primary antibodies, mouse anti-IBV N (4F10), mouse anti-β-actin, mouse anti-Flag, rabbit anti-Src, or rabbit anti-phospho-Src (Tyr416) (Cell Signaling Technology, USA), overnight at 4°C. After washing, membranes were incubated with DyLight 800-conjugated anti-mouse or anti-rabbit IgG secondary antibodies (SeraCare, USA) for 1 h at room temperature. The membranes were visualized using the Odyssey Infrared Imaging System (LI-COR, Bad Homburg, Germany). For quantification of Src activation, band intensities of phospho-Src (p-Src) were normalized to those of total Src using ImageJ software (NIH, USA).

### S1 protein binding assay

CEK and DF-1 cells seeded in 96-well plates were washed three times with PBS and fixed with 4% paraformaldehyde for 15 min at 4°C. Following five washes with PBS, cells were incubated with 50 μL of 15 μg/mL Pro-CS1, Pro-CS1 (H413Q), Pro-LS1, or Pro-LS1 (Q413H) proteins diluted in 1% BSA for 2 h at 4°C. An equal volume of the elution buffer for protein purification was used as a negative control. After five washes with PBS, cells were incubated overnight at 4°C with 50 μL of HRP-conjugated anti-Strep antibody (IBA Life Sciences, Göttingen, Germany) diluted in 1% BSA. Color development was performed using a TMB substrate, and the reaction was stopped with 0.5 M H_2_SO_4_. Absorbance was measured at 450 nm.

### Solid-phase direct glycan binding assay

To evaluate the glycan-binding properties of S1 proteins and viruses, a solid-phase direct binding assay was performed as previously described (86). Briefly, 50 μL of serially diluted (0.313–10 μg/mL) biotinylated 3′-sialyllactose or 6′-sialyllactose in PBS was added to streptavidin-coated high-binding-capacity 96-well plates (Thermo Fisher Scientific) and incubated overnight at 4°C. After washing with PBS, the plates were blocked with 5% skim milk for 1 h at 37°C, followed by washing with PBST (PBS containing 0.05% Tween-20). Subsequently, 50 μL of either purified S1 protein (15 μg/mL) or viruses (5 × 10^8^ viral RNA copies) diluted in 2% skim milk was added to wells and incubated overnight at 4°C. Elution buffer and allantoic fluid from uninfected SPF eggs served as negative controls for the S1 protein and virus, respectively. After washes with PBST, wells were incubated with an HRP-conjugated anti-Strep antibody for 2 h at 4°C to detect bound S1 proteins. For virus detection, plates were incubated with chicken anti-CEA P20 serum (9) for 6 h at 4°C, followed by an HRP-conjugated rabbit anti-chicken antibody for 2 h at 4°C. Subsequently, color development, reaction termination, and absorbance measurement were performed as described in the S1 protein binding assay.

### Statistical analysis

Statistical analysis was performed with GraphPad Prism 8 (GraphPad Software, La Jolla, CA, USA). Data were presented as means ± standard deviations (SD) from three independent biological replicates with technical triplicates. Sample size (*n* = 3 independent experiments) was determined based on established statistical considerations for virological experiments (89). For each experiment, cell culture wells/plates were randomly assigned to different virus treatment groups to minimize selection bias and ensure data reliability. The homogeneity of variances was assessed using the F test (for Student’s *t*-test), the Brown–Forsythe test (for one-way ANOVA), or Levene’s test (for two-way ANOVA). Normality of the data was evaluated using the Shapiro–Wilk test. Statistical significance was determined using unpaired two-tailed Student’s *t*-test, one-way ANOVA with Bonferroni’s multiple comparisons test, or two-way ANOVA with Sidak’s multiple comparisons test. The differences were considered statistically significant if *P* values were <0.05.

## Data Availability

All relevant data are within the article and its supplemental files.

## SUPPLEMENTAL MATERIAL

Figure S1 mbio.00884-26-s0001.tif Replication of LDT3/03 P3 and CEA P23 in Vero cells.

Figure S2 mbio.00884-26-s0002.tif Identification of recovered viruses.

Figure S3 mbio.00884-26-s0003.tif Identification of purified S1 proteins.

Figure S4 mbio.00884-26-s0004.tif Validation of reagent efficacy using NDV.

Figure S5 mbio.00884-26-s0005.tif Effect of replacements the S gene S1 and S2 subunits of LDT3/03 P3 with those from CEA P23 on viral attachment capacity and replication levels in cells.

Table S1 mbio.00884-26-s0006.docx Primers used for construction of plasmids and amplification of CPER fragments.

ASM does not own the copyrights to Supplemental Material that may be linked to, or accessed through, an article. The authors have granted ASM a non-exclusive, world-wide license to publish the Supplemental Material files. Please contact the corresponding author directly for reuse.
