# Supplementary material for: Unique amino acid substitution within the spike glycoprotein S1 subunit promotes infectious bronchitis virus infection via enhancing viral attachment
Source: mBio. 2026 Sep 3;17(10):e00884-26. doi: 10.1128/mbio.00884-26 (PMC13712792; doi:10.1128/mbio.00884-26)
Supplement: Supplementary file 1 [file mbio.00884-26-s0006.docx]

Supplemental Table 1 Primers used for construction of plasmids and amplification of CPER fragments

| **Primer name** | **Primer sequence (5’-3’)** | **Composition** | **Usage** |
| --- | --- | --- | --- |
| G1-F | ACTGAAAATAGATATTAATATATATCTATTGCACTAGC | 1-38 nt of CEA P23 or LDT3/03 P3 genome | Construction of pCEA-G1–G9 and pLDT-G1–G9 |
| G1-R | GCACCAAGTTGTGACATTG | Reverse complement of 2508-2526 nt of CEA P23 or LDT3/03 P3 genome |  |
| G2-F | CTTCCGCTTCAAACGTGAAGAC | 2470-2491 nt of CEA P23 or LDT3/03 P3 genome |  |
| G2-R | CTCTTAAACCTATTAACAGTGCTACTAATAAC | Reverse complement of 7361-7392 nt of CEA P23 or LDT3/03 P3 genome |  |
| G3-F | GTTAGTGGAAAAGAAGGCTGG | 7336-7356 nt of CEA P23 or LDT3/03 P3 genome |  |
| G3-R | CGCATAGTGACAGGGTAAAGTCC | Reverse complement of 9263-9285 nt of CEA P23 or LDT3/03 P3 genome |  |
| G4-F | GCTTGCTCTTATGGTGGTACAG | 9236-9257 nt of CEA P23 or LDT3/03 P3 genome |  |
| G4-R | CAACAGTTGTACACTTTACATCAC | Reverse complement of 10713-10736 nt of CEA P23 or LDT3/03 P3 genome |  |
| G5-F | GTGCTTCCTATAGCCACTGTTC | 10679-10700 nt of CEA P23 or LDT3/03 P3 genome |  |
| G5-R | TAGAAGCTGGCGTATATCAAACATAG | Reverse complement of 13760-13785 nt of CEA P23 or LDT3/03 P3 genome |  |
| G6-F | ACGATTATGATTATTATCGTTATAACAGGC | 13727-13756 nt of CEA P23 or LDT3/03 P3 genome |  |
| G6-R | GTAGCAGCAAGTGCTTTGGTAG | Reverse complement of 17033-17054 nt of CEA P23 or LDT3/03 P3 genome |  |
| G7-F | AGTGGAGTACATCCTGCTTATGC | 17005-17027 nt of CEA P23 or LDT3/03 P3 genome |  |
| G7-R | AATACCCACCAAGAATGCCTCTG | Reverse complement of 20156-20178 nt of CEA P23 or LDT3/03 P3 genome |  |
| G8-F | GTACTGCAGTGAATGCTTCTTCC | 20132-20154 nt of CEA P23 or LDT3/03 P3 genome |  |
| G8-R | CATTAGAGGAATGATACCAAAGCATC | Reverse complement of 23790-23815 nt of CEA P23 or LDT3/03 P3 genome |  |
| G9-F | ATGACTGGTTGTTGTGGTTGTTG | 23762-23784 nt of CEA P23 or LDT3/03 P3 genome |  |
| G9-R | TTTTTTTTTTTTTGCTCTAACTCTATACTAGC | Reverse complement of 27479-27510 nt of CEA P23 genome or 27676-27706 nt of LDT3/03 P3 genome |  |
| G1-F^a^ |  |  | Introdution of silent mutations T2173C into pCEA-G1 |
| CG1-T2173C-R | GTTGAAAACAATGCTGTGCGACACCTTTCAGGACACATAG | Reverse complement of CG1-T2173C-F |  |
| CG1-T2173C-F | CTATGTGTCCTGAAAGGTGTCGCACAGCATTGTTTTCAAC | 2153-2192 nt of CEA P23 genome (The mutation of 2173 nt) |  |
| G1-R^a^ |  |  |  |
| G6-F^a^ |  |  | Introdution of silent mutations T14382A into pLDT-G6 |
| LG6- T14382A-R | CTGGCTTTACGTAAATACCACCTGTAGCTAATACAGTTTCAGAC | Reverse complement of LG6-T14382A-F |  |
| LG6- T14382A-F | GTCTGAAACTGTATTAGCTACAGGTGGTATTTACGTAAAGCCAG | 14361-14404 nt of LDT3/03 P3 genome (The mutation of 14382 nt) |  |
| G6-R^a^ |  |  |  |
| G8-F^a^ |  |  | Construction of pCEA-G8-LS |
| CG8-LS-R | CTAAAAACAGTGACTTCCCCAACAT | Reverse complement of CG8-LS-F |  |
| CG8-LS-F | ATGTTGGGGAAGTCACTGTTTTTAG | 20405-20429 nt of LDT3/03 P3 genome (1-25 nt of LDT3/03 P3 S CDS) |  |
| G8-R^a^ |  |  |  |
| G8-F^a^ |  |  | Construction of pCEA-G8-LS1 |
| CG8-LS1-R | ACGTCTAAAACGACGTGTCTC | Reverse complement of CG8-LS1-F |  |
| CG8-LS1-F | GAGACACGTCGTTTTAGACGT | 22004-22024 nt of LDT3/03 P3 genome (1600-1620 nt of LDT3/03 P3 S CDS) |  |
| G8-R^a^ |  |  |  |
| G8-F^a^ |  |  | Construction of pCEA-G8-LS2 |
| CG8-LS2-R | GCAACTTGTTACATTATCACTAGTAGAAC | Reverse complement of CG8-LS2-F |  |
| CG8-LS2-F | GTTCTACTAGTGATAATGTAACAAGTTGC | 22023-22051 nt of LDT3/03 P3 genome (1619-1647 nt of LDT3/03 P3 S CDS) |  |
| G8-R^a^ |  |  |  |
| G8-F^a^ |  |  | Construction of pCEA-G8-N200S |
| CG8-200-R | GCCAGAACCTTAAATTCTTTCATAATATTATAACTTACAGGTCCACCTGCTTTAAAATAC | Reverse complement of CG8-200-F |  |
| CG8-200-F | GTATTTTAAAGCAGGTGGACCTGTAAGTTATAATATTATGAAAGAATTTAAGGTTCTGGC | 20977-21036 nt of CEA P23 genome (573-632 nt of CEA P23 S CDS) (The mutation of 599 nt) |  |
| G8-R^a^ |  |  |  |
| G8-F^a^ |  |  | Construction of pCEA-G8-H413Q |
| CG8-413-R | CTGGTGGAACGGTGGCTGTTTGTATACGAGAGCCATCGC | Reverse complement of CG8-413-F |  |
| CG8-413-F | GCGATGGCTCTCGTATACAAACAGCCACCGTTCCACCAG | 21624-21662 nt of CEA P23 genome (1220-1258 nt of CEA P23 S CDS) (The mutation of 1239 nt) |  |
| G8-R^a^ |  |  |  |
| G8-F^a^ |  |  | Construction of pCEA-G8-F486L |
| CG8-486-R | CTTCACAGGGATTAACCTTGTAATAATTAAGACCATGTTCACCTTGTACAAC | Reverse complement of CG8-486-F |  |
| CG8-486-F | GTTGTACAAGGTGAACATGGTCTTAATTATTACAAGGTTAATCCCTGTGAAG | 21839-21890 nt of CEA P23 genome (1435-1486 nt of CEA P23 S CDS) (The mutation of 1239 nt) |  |
| G8-R^a^ |  |  |  |
| pCAGGS-N-F | TGTGCGGCCGCATGGCGAGCGTTAAAGCATCTG | 1723-1733 nt of pCAGGS vector + 25961-25982 nt of CEA P23 genome (*Not* I site) | Construction of pCAG-N |
| pCAGGS-N-R | GGCCTCGAGTCAAAGTTCATTTTCACCAAGTGC | Reverse complement of 27167-27190 nt of CEA P23 genome + 1734-1742 nt of pCAGGS vector (*Xho* I site) |  |
| pCAGGS-CS1-F | TGTGCGGCCGCATGTTGGGGAAGTCACTGTTTTTAG | 1723-1733 nt of pCAGGS vector + 20405-20429 nt of CEA P23 genome (*Not* I site) | Construction of pCAG-CS1 and pCAG-CS1-H413Q |
| pCAGGS-CS1-R | GGCCTCGAG*CTTGTCATCGTCATCCTTGTAGTCCTTCTCGAACTGGGGGTGGCTCCA*GCTGCCTCCTCC**CAGGAATGTGCTCAGCAGCACCCATTCGCCATCCTTCCGCACGTAGGCCTGGCCATCTCTAGGGGCCTCAGGGATGTAGCC** ACGTCTAAAACGACGTG | Reverse complement of 22008-22024 nt of CEA P23 genome + **CDS of fibritin trimerization domain** + 1× GGGS linker + *CDS of Strep and Flag* + 1734-1742 nt of pCAGGS vector (*Xho* I site) |  |
| pCAGGS-LS1-F^b^ |  | 1723-1733 nt of pCAGGS vector + 20405-20429 nt of LDT3/03 P3 genome (*Not* I site) | Construction of pCAG-LS1 |
| pCAGGS-LS1-R^c^ |  | Reverse complement of 22008-22024 nt of LDT3/03 P3 genome + **CDS of fibritin trimerization domain** + 1× GGGS linker + *CDS of Strep and Flag* + 1734-1742 nt of pCAGGS vector (*Xho* I site) |  |
| pCAGGS-LS1-F^b^ |  |  | Construction of pCAG-LS1-Q413H |
| pCAGGS-LS1-413R | CTGGTGGAACGGTGGCTGTGTGTATACGAGAGCCATCGC | Reverse complement of pCAGGS-LS1-413F |  |
| pCAGGS-LS1-413F | GCGATGGCTCTCGTATACACACAGCCACCGTTCCACCAG | 21624-21662 nt of LDT3/03 P3 genome (1220-1258 nt of LDT3/03 P3 S CDS) (The mutation of 1239 nt) |  |
| pCAGGS-LS1-R^c^ |  |  |  |
| UTR-F | ATTAAAGGTTAAGGAAGATAGGCATGTAGCTTGATTACCTACATGTCTATCG | 27190-27241 nt of CEA P23 genome or 27387-27438 nt of LDT3/03 P3 genome | Amplification of UTR fragment of CEA P23 and LDT3/03 P3 |
| UTR-R | GTTGTCACTGTCTATTGTATGTCTGCTCACTAAACACCACCAGAACCTG | Reverse complement of F1-F |  |
| F1-F | CAGGTTCTGGTGGTGTTTAGTGAGCAGACATACAATAGACAGTGACAAC | 481-529 nt of CEA P23 or LDT3/03 P3 genome | Amplification of F1 fragment of CEA P23 and LDT3/03 P3 |
| F1-R | GCACCAAGTTGTGACATTGGTGTATAATAAACATTGTCTTCACGTTTGAAGCGG | Reverse complement of F2-F |  |
| F2-F | CCGCTTCAAACGTGAAGACAATGTTTATTATACACCAATGTCACAACTTGGTGC | 2473-2526 nt of CEA P23 or LDT3/03 P3 genome | Amplification of F2 fragment of CEA P23 and LDT3/03 P3 |
| F2-R | CTCTTAAACCTATTAACAGTGCTACTAATAACACCACCAGCCTTCTTTTCCACTAAC | Reverse complement of F3-F |  |
| F3-F | GTTAGTGGAAAAGAAGGCTGGTGGTGTTATTAGTAGCACTGTTAATAGGTTTAAGAG | 7336-7392 nt of CEA P23 or LDT3/03 P3 genome | Amplification of F3 fragment of CEA P23 and LDT3/03 P3 |
| F3-R | CGCATAGTGACAGGGTAAAGTCCTATAACTGTACCACCATAAGAGCAAGC | Reverse complement of F4-F |  |
| F4-F | GCTTGCTCTTATGGTGGTACAGTTATAGGACTTTACCCTGTCACTATGCG | 9236-9285 nt of CEA P23 or LDT3/03 P3 genome | Amplification of F4 fragment of CEA P23 and LDT3/03 P3 |
| F4-R | CAACAGTTGTACACTTTACATCACTTAATTTAGATTGAACAGTGGCTATAGGAAGCAC | Reverse complement of F5-F |  |
| F5-F | GTGCTTCCTATAGCCACTGTTCAATCTAAATTAAGTGATGTAAAGTGTACAACTGTTG | 10679-10736 nt of CEA P23 or LDT3/03 P3 genome | Amplification of F5 fragment of CEA P23 and LDT3/03 P3 |
| F5-R | GAAGCTGGCGTATATCAAACATAGTAGGCCTGTTATAACGATAATAATCATAATCG | Reverse complement of F6-F |  |
| F6-F | CGATTATGATTATTATCGTTATAACAGGCCTACTATGTTTGATATACGCCAGCTTC | 13728-13783 nt of CEA P23 or LDT3/03 P3 genome | Amplification of F6 fragment of CEA P23 and LDT3/03 P3 |
| F6-R | GTAGCAGCAAGTGCTTTGGTAGTTACTGCATAAGCAGGATGTACTCCAC | Reverse complement of F7-F |  |
| F7-F | GTGGAGTACATCCTGCTTATGCAGTAACTACCAAAGCACTTGCTGCTAC | 17006-17054 nt of CEA P23 or LDT3/03 P3 genome | Amplification of F7 fragment of CEA P23 and LDT3/03 P3 |
| F7-R | CACCAAGAATGCCTCTGAGGAAGAAGCATTCACTGCAGTAC | Reverse complement of F8-F |  |
| F8-F | GTACTGCAGTGAATGCTTCTTCCTCAGAGGCATTCTTGGTG | 20132-20172 nt of CEA P23 or LDT3/03 P3 genome | Amplification of F8 fragment of CEA P23 and LDT3/03 P3 |
| F8-R | CATTAGAGGAATGATACCAAAGCATCCACAACAACAACCACAACAACCAGTC | Reverse complement of F9-F |  |
| F9-F | GACTGGTTGTTGTGGTTGTTGTTGTGGATGCTTTGGTATCATTCCTCTAATG | 23764-23815 nt of CEA P23 or LDT3/03 P3 genome | Amplification of F9 fragment of CEA P23 and LDT3/03 P3 |
| F9-R | CGATAGACATGTAGGTAATCAAGCTACATGCCTATCTTCCTTAACCTTTAAT | Reverse complement of UTR-F |  |

^a^ corresponding primers in construction of pCEA-G1–G9 and pLDT3-G1–G9.

^b^ same sequence as pCAGGS-CS1-F primer.

^c^ same sequence as pCAGGS-CS1-R primer.
